# Comprehensive analysis of REST corepressors (*RCOR*s) in pan-cancer

**DOI:** 10.3389/fcell.2023.1162344

**Published:** 2023-06-05

**Authors:** Rong Zheng, Yingying Pan, Xinhui Liu, Feiye Liu, Aimin Li, Dayong Zheng, Yue Luo

**Affiliations:** ^1^ Integrated Hospital of Traditional Chinese Medicine, Southern Medical University, Guangzhou, China; ^2^ Cancer Center, TCM-Integrated Hospital of Southern Medical University, Guangzhou, China; ^3^ Department of Hepatology, TCM-Integrated Hospital of Southern Medical University, Guangzhou, China

**Keywords:** *RCOR*s, pan-cancer, prognosis, biomarker, HCC, cell growth

## Abstract

REST corepressors (*RCOR*s) are the core component of the LSD1/CoREST/HDACs transcriptional repressor complex, which have been revealed differently expressed in various cancers, but the therapeutic and prognostic mechanisms in cancer are still poorly understood. In this study, we analyzed expression, prognostic value, molecular subtypes, genetic alteration, immunotherapy response and drug sensitivity of *RCOR*s in pan-cancer. Clinical correlation, stemness index, immune infiltration and regulatory networks of *RCOR*s in hepatocellular carcinoma (HCC) were detected through TCGA and GSCA database. *In-vitro* experiments were conducted to explore the role of RCOR1 in HCC cells. The expression of *RCOR*s varied among different cancers, and have prognostic values in several cancers. Cancer subtypes were categorized according to the expression of *RCOR*s with clinical information. *RCOR*s were significantly correlated with immunotherapy response, MSI, drug sensitivity and genetic alteration in pan-cancer. In HCC, *RCOR*s were considered as potential predictor of stemness and also had association with immune infiltration. The ceRNA-TF-kinase regulatory networks of *RCOR*s were constructed. Besides, RCOR1 acts as an oncogene in HCC and promotes the proliferation of HCC cells by inhibiting cell cycle arrest and cell apoptosis. Taken together, our study revealed the potential molecular mechanisms of *RCOR*s in pan-cancer, offering a benchmark for disease-related research.

## 1 Introduction

Despite the emerging role of immunotherapy in cancer treatment, cancer is still a major factor which has caused high mortality all over the world, therefore, explorations for more effective anti-tumor therapeutic targets and methods are urgently needed (Sung et al., 2021). In recent years, with the continuous developments of public databases such as The Cancer Genome Atlas (TCGA), more and more oncogenes which might be potential novel molecular targets for cancer therapy have been studied by analyzing their expression and correlation with clinical prognosis and relevant signaling pathways (Blum et al., 2018).

CoREST (REST corepressor), a 66 KD protein with two highly structural conservative SANT domains. It binds to the REST (RE1 silencing transcription factor) to repress the transcription of some neuron-specific genes in non-neuronal cells (Andrés et al., 1999). Recently, CoREST was discovered to be a complex consists of three sub-members: CoREST1 (encoded by *RCOR1*), CoREST2 (encoded by *RCOR2*) and CoREST3 (encoded by *RCOR3*), which functionally related to the programming of cell fate (Saleque et al., 2007; Upadhyay et al., 2014). Each member of this family exhibits unique properties in many physiological processes. *RCOR1* is a co-inhibitor of the transcription factor REST/NRSF, and it interacts with REST to silence the neuron-related gene expression in neural stem cells or non-neural cells (Monaghan et al., 2017) and also regulates hematopoietic differentiation (Yao et al., 2014). *RCOR2* plays a role in maintaining the pluripotency and cortical development of embryonic stem cells (ESCs) by inhibiting the proliferation of ESCs and formation of induced pluripotent stem (iPS) cells (Yang et al., 2011). Both *RCOR1* and *RCOR2* positively influence LSD1 demethylating activity, leading to more active erythro-megakaryocytic differentiation. However, *RCOR*3 serves as a competitor for LSD1 in hematopoietic cells (Upadhyay et al., 2014). In the last few years, researchers have paid extensive attention to the roles that *RCORs* played in cancer. Compared with the control normal groups, *RCOR1* displays different expression level among oral squamous cell carcinoma, prostate cancer, breast cancer, ovarian cancer, lymphoma, glioma patients. *RCOR2* is identified downregulated in emodin-treated lung adenocarcinomas cells (Maimaiti et al., 2022). Except for differential expression, accumulating studies indicated that *RCOR*s probably have some functional effects on initiation and progression of tumors. For example, *RCOR1* directly interacts with MED28 and supresses cancer stem cell (CSC)-like properties in oral cavity squamous cell carcinoma (OCSCC) cells (Xiang et al., 2020). Knock-down of *RCOR1* reflects a gene signature which is associated with novel molecular subtype and predictable for unfavorable PFS of R-CHOP-treated DLBCL patents (Chan et al., 2015). Upregulation of *RCOR1* may maintain the tumor stem-like phenotype in diffuse astrocytoma (DA), anaplastic oligodendroglioma (AO), and glioblastoma multiforme (GBM) (Yucebas et al., 2016). *RCOR1* activates the secretion of angiogenic and inflammatory factors, strengthening tumor-induced angiogenesis and inflammatory responses in breast cancer (Mazumdar et al., 2015). Xue et al. (2011) revealed that serum *RCOR3* level can reflect liver injury degree and it is a biomarker of intrahepatic cholangiocarcinoma (Lv et al., 2017). In addition, *RCOR3* shows lower expression in colorectal cancer patients, with hypermethylation on promoters (Liu et al., 2019). However, the underlying therapeutic and prognostic mechanisms of *RCOR*s in cancer remain unclear.

In this study, we detected the expression and prognostic values of *RCOR*s in pan-cancer, as well as the association between *RCOR*s expression and cancer subtypes, responsiveness to immunotherapy, signaling pathway, drug sensitivity and genetic alteration. Besides, correlation analysis of *RCOR*s expression with clinicopathological parameters, stemness signature, immune infiltrations and regulatory networks were completed in HCC. Additionally, we identified RCOR1 is upregulated in HCC, and promotes cell proliferation by inhibiting cell cycle arrest and cell apoptosis. Generally, our study revealed the potential molecular mechanisms of *RCORs* in pan-cancer, offering a benchmark for cancer-related research.

## 2 Materials and methods

### 2.1 TCGA data acquisition and processing

The data of 11,069 tumor patients across 33 different tumor types was downloaded from TCGA database (https://portal.gdc.cancer.gov/), which contains transcriptome RNAseq data, survival information and clinicopathological characteristics including age, sex, and tumor stage. Abbreviations of the 33 tumor types were provided in Supplementary Table S1. All expression data were coded as fragments per kilobase per million (FPKM) and normalized by log2 transformation. R software and its packages were used to process all these data.

### 2.2 Expression of *RCOR*s in pan-cancer

Expression profile of *RCOR*s in tumor tissues, adjacent non-tumor tissues and tumor cell lines were extracted from TCGA database. *RCOR*s expression in normal tissues was acquired from the Human Protein Altas database (https://www.proteinatlas.org/), where RNA-seq tissue data was generated from Genotype-Tissue Expression (GTEx). Differential expression studies were performed between tumor and adjacent non-tumor tissues by Wilcox test.

### 2.3 Prognostic value analysis of *RCORs* in pan-cancer

Univariate cox proportional hazards model and Kaplan-Meier method were applied to assess the relationship between *RCOR*s expression and clinical outcomes, including overall survival (OS), progression-free survival (PFS), disease-free survival (DFS) and disease-specific survival (DSS). Hazard ratios (HRs) were calculated by Cox model, with 95% confidence intervals (95% CI). Schoenfeld’s residuals test was used to perform proportional hazards (PH) hypothesis on risk models. Log-rank test was used for Kaplan-Meier survival analysis.

### 2.4 Molecular subtype models in pan-cancer

Non-negative matrix factorization (NMF) clustering algorithm was used to categorize patients into different subtypes (Yuan et al., 2020). Differences in clinicopathological characteristics including sex, age, tumor stage and etc., of each subtype among cancers were compared, and the clinical data were processed and analyzed by the “CreateTableOne” function within R package “TableOne” (https://github.com/agapiospanos/TableOne). Details for the parameter setting in statistical analysis were displayed in Supplementary Table S2.

### 2.5 Pathway activity and drug sensitivity analysis in pan-cancer

GSCALite (http://bioinfo.life.hust.edu.cn/web/GSCALite/) is an online platform for genomic cancer research which contains 33 cancers’ data, and GSCA (http://bioinfo.life.hust.edu.cn/GSCA/#/) is the updated version of GSCALite (Liu et al., 2023). Correlation of *RCOR*s expression with 10 common cancer-related signaling pathways (TSC/mTOR, RTK, RAS/MAPK, PI3K/AKT, Hormone ER, Hormone AR, EMT, DNA Damage Response, Cell Cycle, Apoptosis) were conducted through GSCALite. Student’s t test was performed in this module. Anti-tumor drug sensitivity was analyzed using the “Drug” module of GSCA by Pearson test. *p*-value was adjusted by FDR.

### 2.6 Responsiveness to immunotherapy and genetic alterations analysis of *RCOR*s in pan-cancer

Spearman test was used to analyze the correlation between *RCOR*s expression and Tumor Mutation Burden (TMB) and Microsatellite instability (MSI) respectively. Genetic alteration of *RCOR*s and its correlation with prognosis (OS, PFS, DFS, and DSS) in pan-cancer were investigated using the cBioPortal (http://www.cbioportal.org/).

### 2.7 *RCOR*s in HCC

Correlation between *RCOR*s expression and clinical features, immune checkpoint genes in HCC was utilized based on TCGA data. Stemness models were established, multivariate Cox proportional hazards regression was used to evaluate the association of clinical features, stemness indices with OS and Kruaskal-Wallis analysis was used to examine the of stemness indices among different HCC subtypes. GSCA was employed to analyze the correlation between *RCOR*s expression and immune infiltration in HCC. Upstream miRNAs of *RCOR*s were searched from mirecords (https://mirecords.biolead.org/), mirtarbase (https://mirtarbase.cuhk.edu.cn/) and tarbase (https://dianalab.e-ce.uth.gr/). MiRNA-affiliated lncRNAs were retrieved by R package “multiMiR” (Ru et al., 2014). Competing endogenous RNA (ceRNA) networks were constructed, eliminating mRNAs which were unrelated to *RCOR*s. Transcription factors (TFs) of HCC were downloaded from AnimalTFDB3.0 (http://bioinfo.life.hust.edu.cn/AnimalTFDB#!/). Pearson correlation coefficients of *RCOR*s-affiliated TFs were calculated by setting threshold |cor| ≥ 0.7 and *p* ≤ 0.05. The upstream kinases of *RCOR*s were predicted by using X2Kgui. All networks were mapped by Cytoscape.

### 2.8 Tissue microarrays and immunohistochemistry

Tissue Microarray (TMA) were obtained from Shanghai Outdo Biotech Co., Ltd. (Cat No: HLivH180Su18; Shanghai, China) with 90 cases of HCC tissues and adjacent non-tumor tissues. After deparaffinization and rehydration, using microwave treatment to perform heat-mediated antigen retrieval with a pH 9.0 Tris/EDTA buffer. Incubating tissues in 3% H_2_O_2_ at room temperature for 15 min to block endogenous peroxidase activity and 30 min’ incubation of goat serum (Zhongshan Golden Bridge Biotechnology Co., Ltd., Beijing, China) at 37°C to block non-specific staining. A rabbit anti-human RCOR1 mAb (1:50, ab183711; Abcam, United States) with overnight incubation at 4°C, followed by incubation with a secondary anti-rabbit/mouse HRP-conjugated antibody (Zhongshan Golden Bridge Biotechnology Co., Ltd., Beijing, China) at room temperature for 1 h. Subsequently, TMA was washed by phosphate-buffered saline (PBS) for 3 times, and colorized with DAB-positive substrate. The slides were counterstained with hematoxylin. Staining intensity was classified into 4 grades: 0 (negative), 1 (weak), 2 (moderate), and 3 (strong). The percentage of staining-positive cells was scored as 0 (0%), 1 (1%–10%), 2 (11%–50%), 3 (51%–80%), and 4 (81%–100%). The overall score was calculated using the following formula: overall score = intensity score × percentage score. The total scores of 0–4, 5–8, and 9–12 were defined as weak positive, moderate positive, and strong positive, respectively (Scharl et al., 1990).

### 2.9 Cell culture and transfection

Four HCC cell lines: SK-Hep1, Hep3B, Huh7, and HCCLM3 were obtained from TCM-Integrated Hospital of Southern Medical University (Guangzhou, China). Cells were routinely maintained in high-glucose DMEM (Gibco Co., Ltd., Grand Island, NY, United States) and supplemented with 10% fetal bovine serum (Gibco) at 37°C with 5% CO_2_. *RCOR1* siRNAs and negative control (NC) siRNA were purchased from RIBOBIO (Guangzhou, China). The *RCOR1* overexpression and negative control plasmids were purchased from OBIO (Shanghai, China). Hep3B was used for loss-of-function studies and Huh7 was used for gain-of-function studies. Lipofectamine 3,000 and P3000 (Invitrogen Life Technologies, Carlsbad, CA, United States) were used to transfect cells with siRNAs and plasmids. Three siRNA-targeted sequences (provided by RIBOBIO) as follows were used to silence *RCOR1*: si-1: 5′-CCA​GAT​AAA​TCT​ATA​GCA​A-3′; si-2: 5′-GCA​TGG​GTA​CAA​CAT​GGA​A-3′; si-3: 5′-GGC​AGA​ACA​TGG​TAA​AGA​A-3′. Cells were seeded in 6-well plates and transfected for 48 h, then harvested for further assays.

### 2.10 Real-time quantitative PCR

Total RNA was isolated with Trizol reagent (#YY101, EpiZyme Biotech Co., Ltd., Shanghai, China). 1 μg total RNA was reversely transcribed into cDNA using PrimeScript RT Master Mix (#RR036A, Takara Biotechnology (Dalian) Co., Ltd., China) according to the manufacturers’ protocols. TB Green™ Premix Ex Taq™ II (#RR820A, Takara Biotechnology (Dalian) Co., Ltd., China) was used as the fluorescent dye. Real-time (RT) PCR was performed by LightCycler480 system (Roche, Switzerland) with the *RCOR1*-specific primers as follows: forward, 5′-CGA​GGA​CTA​AAA​CTA​GTG​TGA​TGG-3′, reverse, 5′-TGC​CTC​TTC​CAG​TTC​ATC​CT-3′; *β-actin*: forward, 5′-GGG​AAA​TCG​TGC​GTG​ACA​TTA​AGG-3′, reverse, 5′-CAG​GAA​GGA​AGG​CTG​GAA​GAG​TG-3′. The *β-actin* gene was used as the housekeeping control gene. Relative gene expression was calculated using the 2^^−ΔΔct^ method. All the oligonucleotide primers were synthesized from Invitrogen.

### 2.11 Western blotting

RIPA lysis buffer (#PC101, EpiZyme Biotech; Shanghai, China) was used to extract total protein. It was supplemented with protease inhibitors (#GRF101, EpiZyme Biotech) and phosphatase inhibitors (#GRF102, EpiZyme Biotech), separated by SDS-PAGE, and transferred to a PVDF membrane (Millipore, Billerica, MA). The membranes were blocked with 5% non-fat milk at room temperature for 1 h. The membranes were incubated with primary antibody at 4°C overnight. Tris-HCl solution with Tween-20 (TBST) was used to wash the membranes three times for 10 min. Subsequently, they were incubated with horseradish peroxidase-conjugated secondary antibody (goat-anti-rabbit/mouse IgG, LI-COR, United States) for 1 h at room temperature. Protein bands were visualized by using the enhanced chemiluminescence (ECL) Plus kit (Millipore). The membranes were detected by the Odyssey infrared imaging system (LICOR, United States). The primary antibodies are as follows: anti-CoREST (RCOR1) rabbit-mAb (1:1,000, #14567T; CST, United States), anti-β-actin mouse mAb (1:5,000, A5316; Sigma, United States).

### 2.12 Cell viability analysis

SiRNAs and overexpression plasmids which were used to transfect HCC cells were seeded into 96-well plates at a density of 1,500 cells per well in triplicate and incubated at 37°C in 5% CO_2_. After 6 h, 24 h, 48 h, 72 h, and 96 h of cultivation, 10 µL CCK-8 (Dojindo, Kumamoto, Japan) reagent was added to each well, followed by incubating for 2.5 h at 37°C in 5% CO_2_. The absorbance at 450 nm was measured using a microplate reader (BioRad, Berkeley, CA, United States).

### 2.13 5-Ethynyl-2′-deoxyuridine (EDU) assay

Cells were seeded in serum-free media for 6 h prior to treatment to allow for cell cycle synchronization. After 48 h transfection of siRNAs or overexpression plasmids, cells were pulsed with Cell-Light EdU buffer (Apollo567 *In Vitro* Kit; #C10310-1; RIBOBIO, Guangzhou, China) and incubated for 2 h before fixation in 4% paraformaldehyde (PFA) for 15 min. The results were imaged under an inverted fluorescence microscope. Quantification of S phase cells was operated using ImageJ. The fraction of S phase cells for each field of view was captured and analyzed.

### 2.14 Cell cycle analysis

5 × 10^4 cells were seeded into each well of six-well plates. After transfection with siRNAs or plasmids, cells were cultured for 48 h, then collected and fixed with 75% ethanol overnight at −20°C. The fixed cells were washed with cold PBS and suspended in 500 μL PBS containing RNAase and stained in the dark with propidium iodide at room temperature for 30 min. Cell suspension was subjected to FACS (BD Co., San Jose, CA, United States) to analyze the percentage of cells at G1, S and G2/M phases of the cell cycle.

### 2.15 Apoptosis analysis

Cells were stained with Annexin V-FITC and propidium iodide kit (AO 2001-02P-G, Tianjin Sungene Biotech Co., Ltd., Tianjin, China) to evaluate apoptosis by flow cytometry. 5 × 10^4 cells were washed twice with PBS and stained with 5 µL Annexin V-FITC and 5 µL propidium iodide solution in 1 × binding buffer for 15 min at room temperature in the dark. Additional 400 µL of 1 × binding buffer was added to the cell suspension. Apoptosis rates were determined by flow cytometry (BD).

### 2.16 Statistical analysis

Statistical methods which were used in bioinformatic validations are shown above. For *in-vitro* validation data, values are presented as the mean ± SD for three independent experiments. All statistical analysis were performed and imaged with GraphPad Prism 8.0 software (GraphPad, La Jolla, CA, United States). Differences between groups were analyzed by Student’s *t*-test for two groups. The median expression level of RCOR1 was used as the cut-off for the high and low RCOR1 group in IHC scoring assessment. Test level was set at both sides α = 0. 05, *p <* 0.05 was considered statistically significant. Significant differences in figures and tables are marked as follows: *p < 0.05, **p < 0.01, and ***p < 0.001.

## 3 Results

### 3.1 Pan-caner expression lanscape of *RCOR*s

**FIGURE 1:**
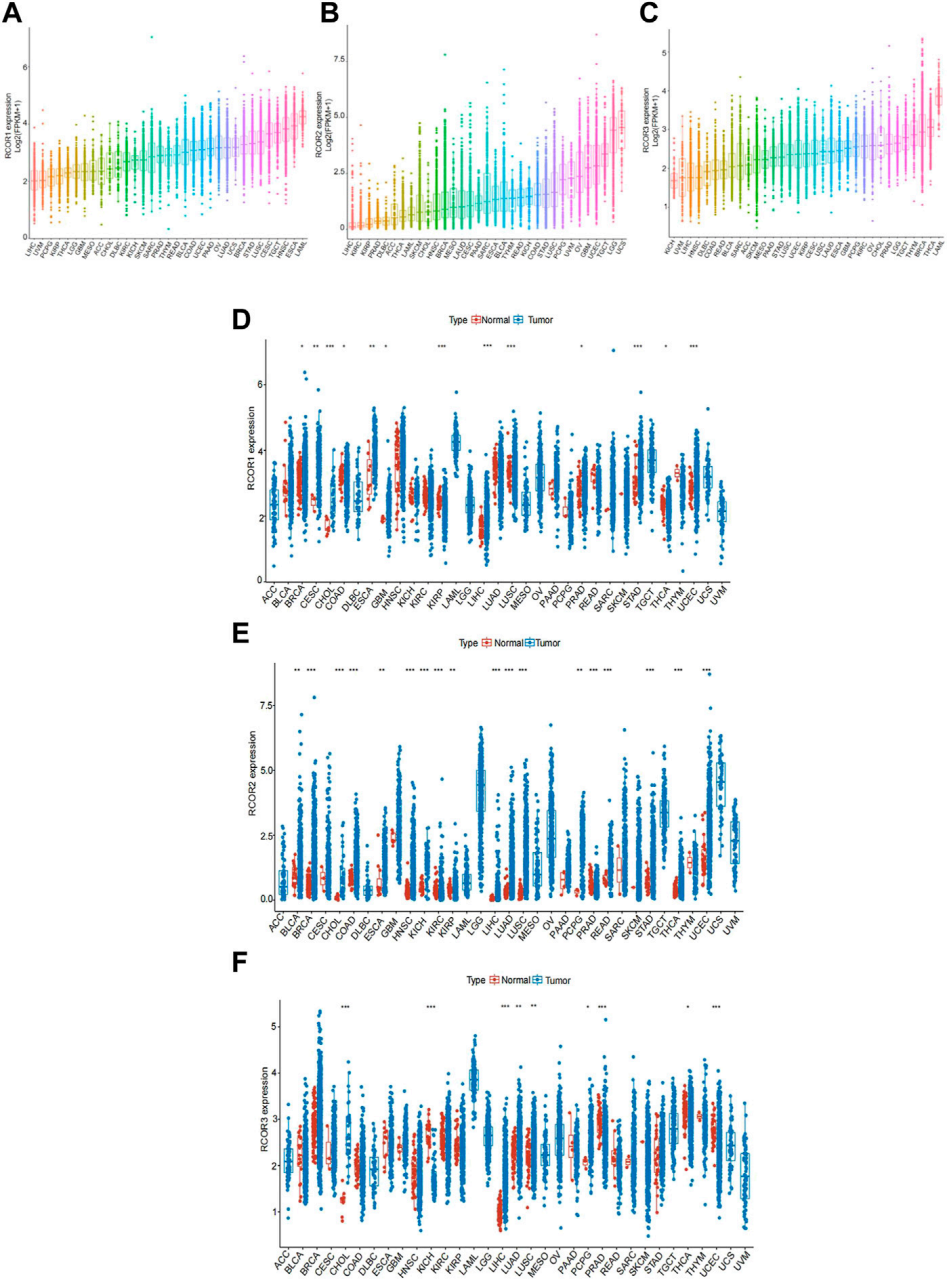
Pan-caner expression lanscape of *RCORs*. **(A)** *RCOR1*, **(B)** *RCOR2* and **(C)** *RCOR3* expression levels among different cancer tissues. **(D)** *RCOR1*, **(E)** *RCOR2* and **(F)** *RCOR3* expression in different cancer tissues (blue) and adjacent non-tumor tissues (red). *p < 0.05, **p < 0.01, and ***p < 0.001.

Gene expression analyses of *RCOR*s in pan-cancer were employed. As shown in Figures 1A–C **,** the expression of *RCOR*s in various cancer tissues were analyzed and the expression levels of all *RCOR* family members in LIHC were relatively low compared to other cancer types. Besides, both *RCOR1* and *RCOR3* displayed highest expression in LAML, whereas *RCOR2* was obviously upregulated in UCS and LGG, indicating that *RCOR*s expression may varied among different cancer types. Consistently, expression of *RCOR*s also varied among multiple normal samples and tumor cell lines (Supplementary Figures S1A–C, S2A–C). Further differential expression analyses of *RCOR*s expression between tumor tissues and adjacent non-tumor tissues were also performed, we found that *RCOR1* and *RCOR2* are significantly higher in tumor than in adjacent non-tumor tissues in BRCA, CHOL, COAD, ESCA, KIRP, LIHC, LUAD, PRAD, STAD, THCA, and UCEC (*p* < 0.05, Figures 1D, E) and *RCOR3* was highly expressed in most cancer tissues except KICH (Figure 1F). These results revealed that the expression of *RCOR*s varies among different cancers and normal tissues and differentially expressed between tumor tissues and adjacent non-tumor tissues, indicating that *RCOR*s may be potential cancer biomarkers.

### 3.2 Prognostic value of *RCOR*s in pan-cancer

**FIGURE 2:**
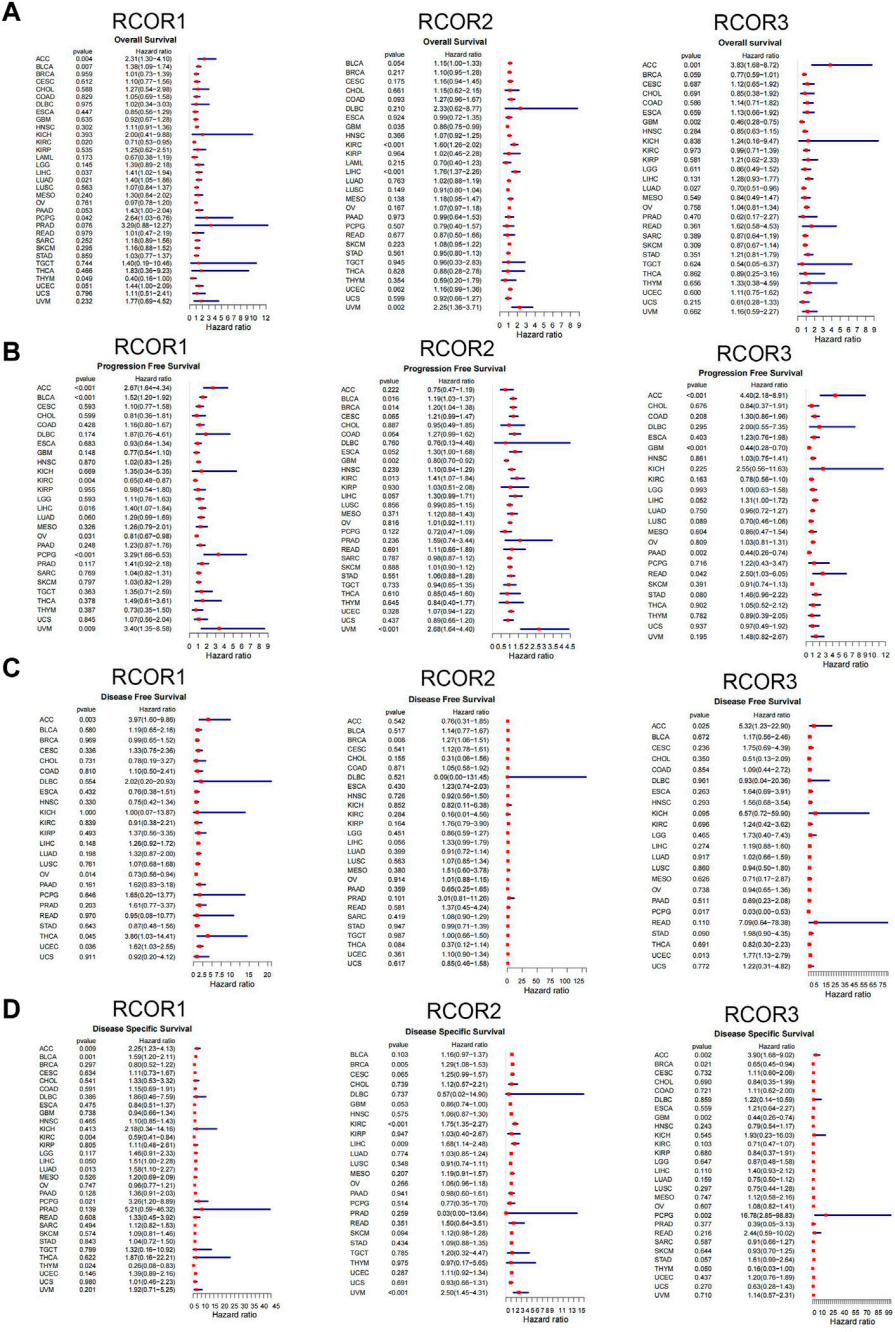
Univariate cox proportional hazards model of *RCOR*s in pan-cancer. Forest maps for OS **(A)**, PFS **(B)**, DFS **(C)** and DSS **(D)** with hazard ratios (log10) and 95% confidence intervals from TCGA; HR < 1 represents low risk and HR > 1 represents high risk.

Univariate Cox models were utilized to estimate the association between the *RCOR*s expression and the prognosis of patients in pan-cancer, Schoenfeld’s residuals test was used to perform proportional hazards (PH) hypothesis on risk models, and the negative results for PH test were showed in Supplementary Figures S3–S6. The survival metrics included OS, PFS, DFS, and DSS. As shown in Figure 2A, univariate Cox regression analysis of the results from 33 types of cancer revealed that *RCOR1* expression was unfavorably associated with OS of ACC (HR = 2.31, 95% CI 1.30–4.10), BLCA (HR = 1.38, 95% CI 1.10–1.74), LIHC (HR = 1.41, 95% CI 1.02–1.94), LAUD (HR = 1.40, 95% CI 1.05–1.86) and PCPG (HR = 2.64, 95% CI 1.03–6.76) (*p* < 0.05). Upregulation of *RCOR2* asscociated to poor OS of KIRC (HR = 1.60, 95% CI 1.26–2.02), LIHC (HR = 1.76, 95% CI 1.37–2.25) and UVM (HR = 2.25, 95% CI 1.36–3.71) (*p* < 0.05). High *RCOR3* expression level was correlated with poor OS of ACC (HR = 3.83, 95% CI 1.68–8.72) (*p* < 0.05).

As shown in Figure 2B, increased *RCOR1* expression was associated with better PFS in KIRC (HR = 0.65, 95% CI 0.48–0.87) and OV (HR = 0.81, 95% CI 0.67–0.98) (*p* < 0.05). High *RCOR2* expression was correlated with better PFS in GBM (HR = 0.80, 95% CI 0.70–0.92). Conversely, upregulated *RCOR3* expression was correlated to poor PFS of ACC (HR = 4.40, 95% CI 2.18–8.91) and READ (HR = 2.50, 95% CI 1.03–6.05) (*p* < 0.05). In Figure 2C, *RCOR1* expression has association with DFS in ACC, OV and UCEC (*p <* 0.05). *RCOR2* could serve as a risk factor for BRCA (HR = 1.27, 95% CI 1.06–1.51) (*p <* 0.05), and DFS-related hazard ratios for *RCOR3* expression were significant in PCPG and UCEC (*p <* 0.05). In Figure 2D, *RCOR1* expression was significantly related to DSS in ACC, BLCA, LAUD, PCPG and THYM (*p <* 0.05). *RCOR2* expression was associated with DSS in BRCA, KIRC, LIHC, and UVM (*p <* 0.05). *RCOR3* expression was significantly correlated with DSS in ACC, BRCA, and GBM (*p <* 0.05).

**FIGURE 3:**
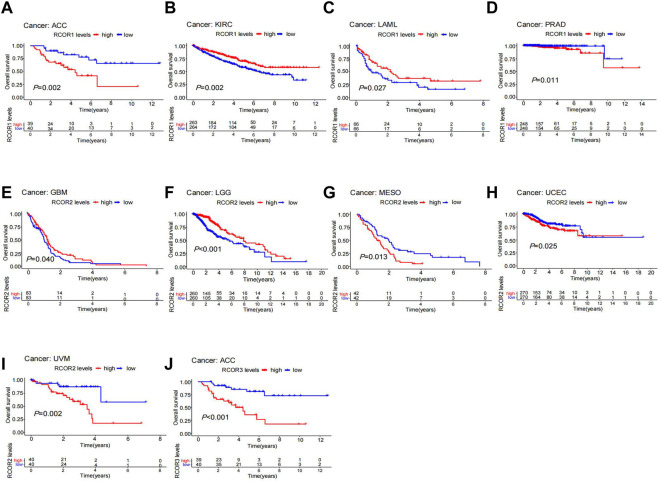
Kaplan-Meier survival analysis of *RCOR*s in different cancers. *RCOR1* as a potential prognosis factor in ACC **(A)**, KIRC **(B)**, LAML **(C)** and PRAD **(D)**; *RCOR2* as a potential prognosis factor in LGG **(E)**, MESO **(F)**, UCEC **(G)**, GBM **(H)** and UVM **(I)**; *RCOR3* as a potential prognosis factor in ACC **(J)**.

On the other hand, Kaplan-Meier analyses were used to further assess the association between *RCOR*s expression and cancer prognosis. *RCOR1* expression positively associated with OS of KIRC and LAML. On the contrary, it negatively related to OS of ACC and PRAD (*p <* 0.05, Figures 3A–D). Higher expression of *RCOR2* indicated poor OS in MESO, UCEC, and UVM, whereas it suggested longer OS in GBM and LGG (*p <* 0.05, Figures 3E–I). *RCOR3* expression was correlated to prognosis of ACC (*p <* 0.05, Figure 3J). PFS, DFS and DSS in 33 tumors were also analyzed, and the results with significance can be seen in Supplementary Figures S7–S8. All the above data revealed *RCOR*s family was related to prognosis and may serve as cancer biomarkers.

### 3.3 Analysis between *RCOR*s and cancer molecular subtypes

**FIGURE 4:**
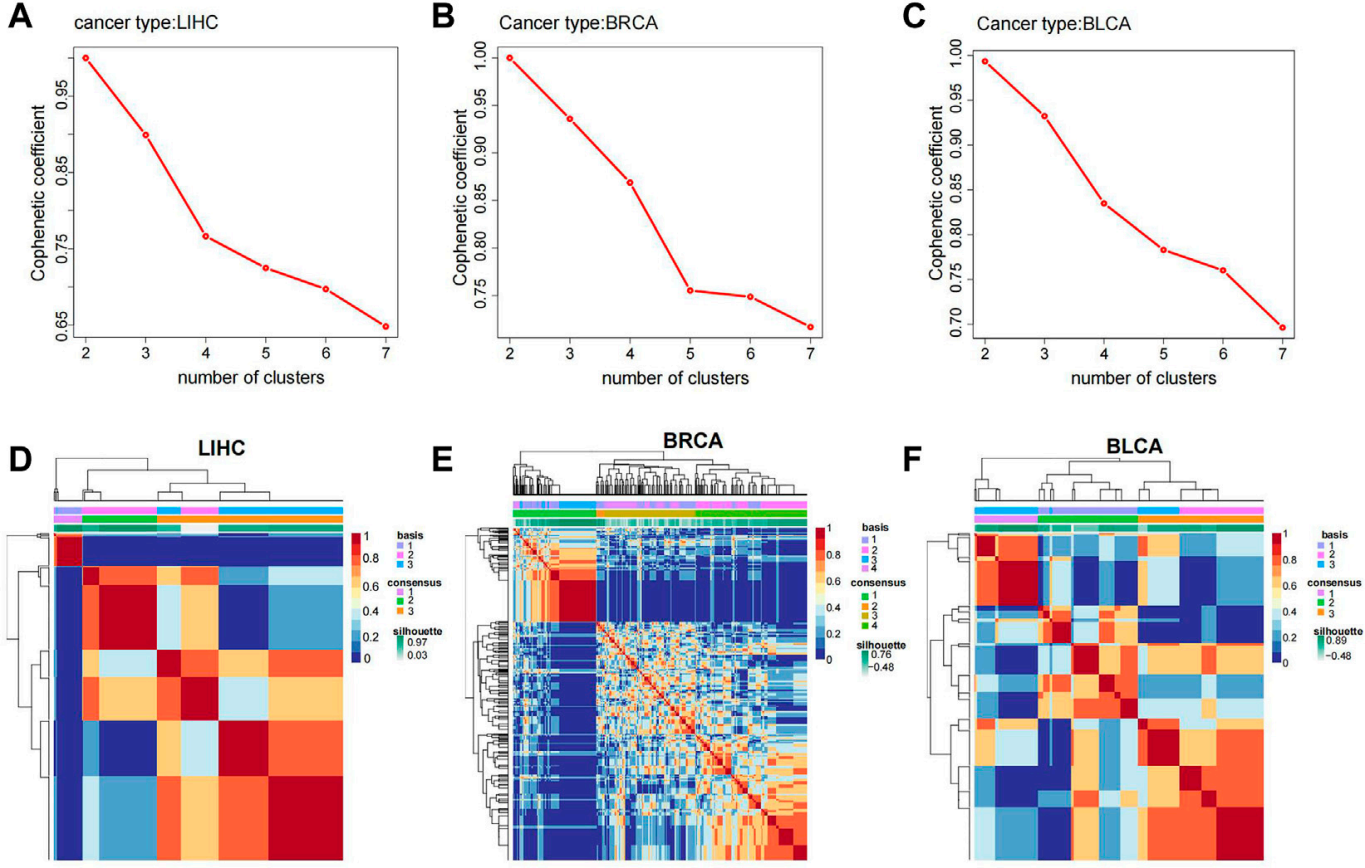
Molecular subtype analysis in various cancers. Cophenetic coefficient in *RCOR*s in HCC **(A)**, BRCA **(B)** and BLCA **(C)**; heatmaps of molecular subtypes of *RCOR*s in HCC **(D)**, BRCA **(E)** and BLCA **(F)**.

Based on the expression of *RCOR* members across multiple cancers, we divided cancer samples into different molecular subtypes through non-negative matrix factorization (NMF) clustering algorithm and R package “NMF” was used for classification. Setting a rank = 2:7 as the basis of classification, the criteria was to recognize the point which locate at the ahead of the largest decline of the cophenetic curve. As the cophenetic curve represented, HCC patients were divided into 3 subtypes (Figures 4A, D). BRCA patients were divided into 4 subtypes (Figures 4B, E) and BLCA patients were divided into 3 subtypes (Figures 4C, F) using the same approach. Interestingly, correlations between molecular subtypes and clinicopathological characteristics were discussed, significant difference was found in M-stage and TNM stage among different subtypes in HCC (Table 1). In addition, different age distributions were also found in different subtypes in BRCA and BLCA (Supplementary Tables S3–S4). Taken together, different cancer molecular subtypes based on *RCOR*s expression exhibit different clinicopathological features.

**TABLE 1:** Correlation between clinicopatological parameters and different molecular subtypes in HCC.

	Subtypes			*p*-value
	Ⅰ	Ⅱ	Ⅲ	
Number	35	146	190	—
Gender				
Male (%)	25 (71.4)	91 (62.3)	134 (70.5)	0.245
Female (%)	10 (28.6)	55 (37.7)	56 (29.5)	
Age (mean ± SD)	61.57 (±10.73)	58.60 (±13.54)	59.70 (±13.96)	0.471
T stage (%)				
T0	0 (0.0)	0 (0.0)	1 (0.5)	0.31
T1	10 (28.6)	76 (52.1)	95 (50.3)	
T2	13 (37.1)	40 (27.4)	41 (21.7)	
T3	11 (31.4)	26 (17.8)	43 (22.8)	
T4	1 (2.9)	4 (2.7)	8 (4.2)	
Tx	0 (0.0)	0 (0.0)	1 (0.5)	
M_stage (%)				
M0	29 (82.9)	114 (78.1)	123 (64.7)	0.011^*^
M1	0 (0.0)	3 (2.1)	1 (0.5)	
MX	6 (17.1)	29 (19.9)	66 (34.7)	
N_stage (%)				
N0	23 (65.7)	104 (71.2)	125 (66.1)	0.702
N1	1 (2.9)	1 (0.7)	2 (1.1)	
NX	11 (31.4)	41 (28.1)	62 (32.8)	
stage (%)				
I	9 (25.7)	72 (49.3)	90 (47.4)	0.043^*^
II	12 (34.3)	39 (26.7)	36 (18.9)	
III	14 (40.0)	32 (21.9)	61 (32.1)	
IV	0 (0.0)	3 (2.1)	3 (1.6)	
group (%)				
1	35 (100.0)	0 (0.0)	0 (0.0)	<0.001^***^
2	0 (0.0)	146 (100.0)	0 (0.0)	
3	0 (0.0)	0 (0.0)	190 (100.0)	

### 3.4 Correlation of *RCOR* family with responsiveness to immunotherapy, drug sensitivity and signaling pathways

**FIGURE 5:**
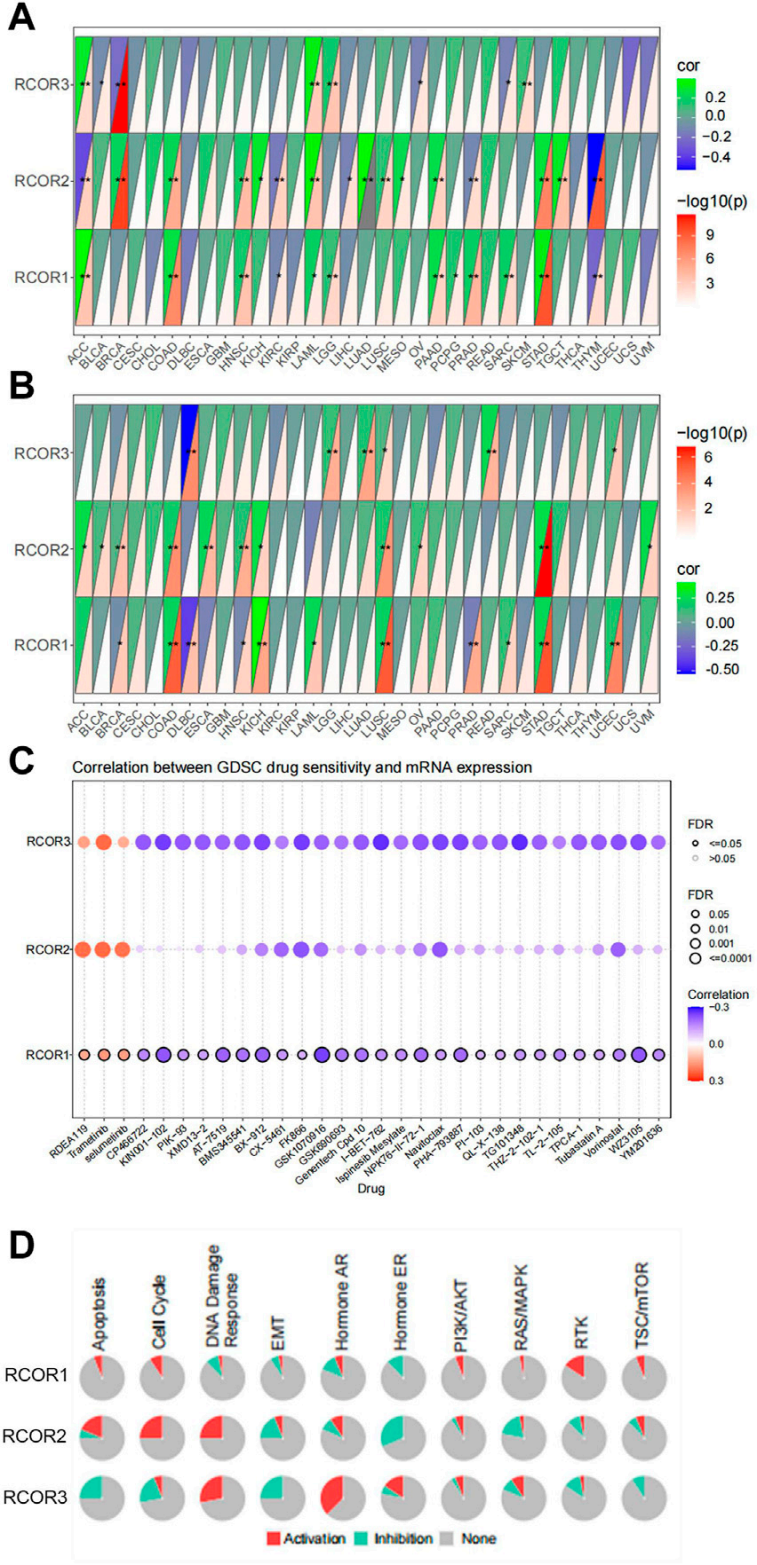
Pan-cancer analysis of *RCOR*s in responsiveness to immunotherapy, drug sensitivity and signaling pathway. Correlation of *RCOR*s expression with TMB **(A)**, MSI **(B)**. Drug sensitivity analysis **(C)** and proportion of activation or inhibition of signaling pathway **(D)** involved in GSCALite. *p < 0.05, **p < 0.01, and ***p < 0.001.

TMB (Tumor Mutation Burden) and MSI (Microsatellite Instability) are two novel biomarkers that relevant to responsiveness to immunotherapy. Spearman analysis was used to analyze the correlation of *RCOR*s expression with TMB and MSI. The results were displayed in Figures 5A, B respectively. According to our results, *RCOR1* expression was positively associated with TMB in ACC, COAD, HNSC, LAML, LGG, PAAD, PCPG, PRAD, SARC, STAD, whereas it negatively associated with TMB in KIRC and THYM. *RCOR2* expression positively connected with TMB in BRCA, COAD, HNSC, KICH, LAML, LAUD, LUSC, MESO, PAAD, STAD, and TGCT, but adverse correlations were obtained from ACC, KIRC, LIHC, PRAD, and THYM. Expression of *RCOR3* was positively correlated with TMB in ACC, LAML, LGG, and SKCM, it was inversely linked with TMB in BLCA, BRCA, OV, SARC. For MSI, positive correlations with *RCOR1* expression were illustrated in COAD, KICH, LAML, LUSC, SARC, STAD, and UCEC, opposite effects were found in BRCA, DLBC, HNSC, and PRAD. Only positive correlations were found with *RCOR2* in ACC, BLCA, BRCA, COAD, ESCA, KICH, HNSC, LUSC, OV, STAD, and UVM. Positive connections between *RCOR3* and MSI were displayed in LGG, LAUD, LUSC, READ, and UCEC, whereas it was negative in DLBC.

Furthermore, we conducted anti-tumor drug sensitivity analysis through the online database GSCA. Correlation analysis between gene expression and IC50 of drugs was performed by Pearson test. We selected the top 30 ranked anti-tumor drugs to show their correlations with *RCOR*s expression, as shown in Figure 5C, It was concluded that RDEA119, Trametinib and Selumetinib positively associated with all *RCOR* members. However, no statistical significance appeared with *RCOR2* among CP466722, KIN001-102, PIK93, and XMD13-2. Complete information for drugs study can be seen in Supplementary Table S5.

Finally, GSCALite website was used for speculating the potential roles of *RCOR*s in 10 classic signaling pathways (TSC/mTOR, RTK, RAS/MAPK, PI3K/AKT, Hormone ER, Hormone AR, EMT, DNA Damage Response, Cell Cycle, Apoptosis), which are considered extensively involved in the process of tumorgenesis. *RCOR1* showed obvious activation of RTK and Cell Cycle, whereas it significantly correlated with inhibition of Hormone ER. Significant correlations were found between *RCOR2* and activation of Cell Cycle and Apoptosis. *RCOR2* had similar inhibition effect of Hormone ER as *RCOR1. RCOR3* remarkably correlated with activation of Hormone AR and DNA Damage Response and inhibition of EMT (Figure 5D).

### 3.5 Analysis of genetic alteration of *RCOR*s in pan-cancer

**FIGURE 6:**
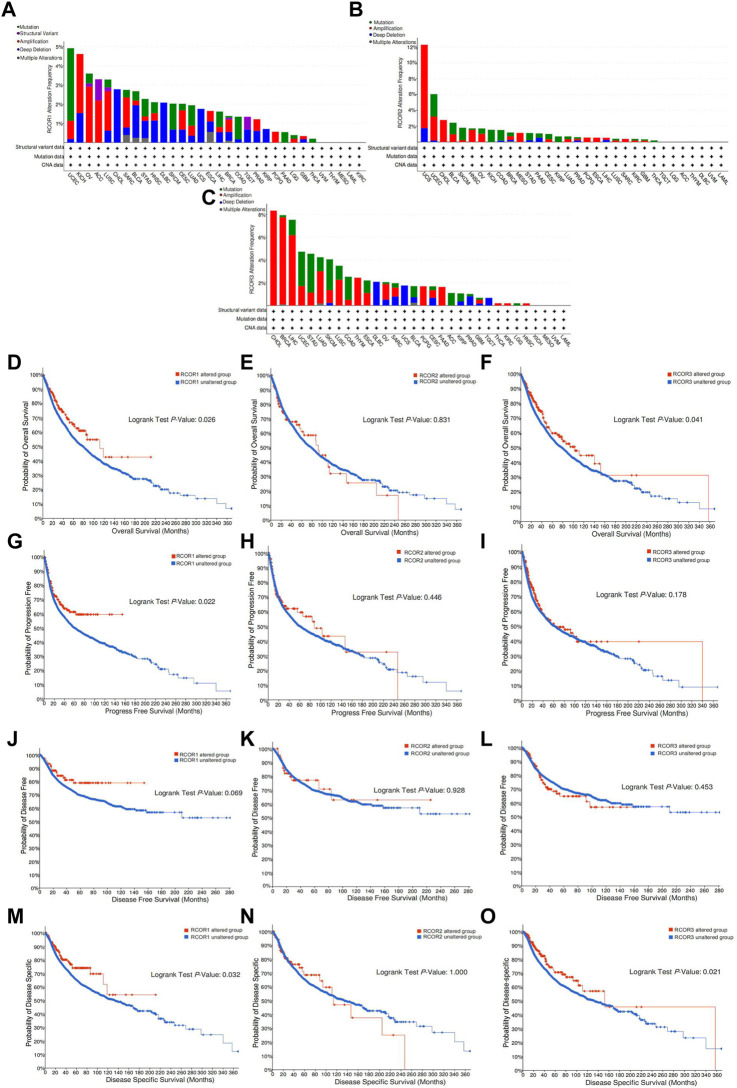
Genetic alteration of *RCOR*s in pan-cancer. The alteration frequency of *RCOR1* **(A)**, *RCOR2* **(B)** and *RCOR3* **(C)** in different cancers. Effect of *RCOR1* **(D)**, *RCOR2* **(E)** and *RCOR3* **(F)** alteration status on OS. Effect of *RCOR1* **(G)**, *RCOR2* **(H)** and *RCOR3* **(I)** alteration status on PFS. Effect of *RCOR1* **(J)**, *RCOR2* **(K)** and *RCOR3* **(L)** alteration status on DFS. Effect of *RCOR1* **(M)**, *RCOR2* **(N)** and *RCOR3* **(O)** alteration status on DSS.

cBioPortal (TCGA PanCancer Atlas) was employed to analyze genetic alterations in *RCOR* family. Genetic alterations of *RCOR1* were detected in 28 cancer types, among them UCEC and KICH had higher alteration frequency (>4%). “Mutation” was the dominant type of alteration of UCEC, and “amplification” was the primary alteration form of KICH (Figure 6A). The highest gene alteration frequency of *RCOR2* (>12%) occurred in UCSC, revealing that “amplification” is the primary type of alteration (Figure 6B). For *RCOR3*, patients with CHOL, BRCA and LIHC had relatively obvious alteration frequency (>4%), “amplification” was also the most common type alteration (Figure 6C). In addition, effects of *RCOR*s alteration status on cancer prognosis were also covered. For *RCOR1* and *RCOR3*, altered groups correlated with better OS (*p <* 0.05) (Figures 6D–F). Figures 6G–I displayed that altered *RCOR1* positively linked with PFS (*p <* 0.05), whereas no significance appeared in altered *RCOR2* and *RCOR3*. Altered *RCOR*s did not show significant effects on DFS compared to unaltered groups (Figures 6J–L). Figures 6M–O indicated that *RCOR1* and *RCOR3* alteration had positive associations with DSS (*p <* 0.05).

### 3.6 *RCOR*s were significantly correlated with age, sex and tumor stage of HCC

**FIGURE 7:**
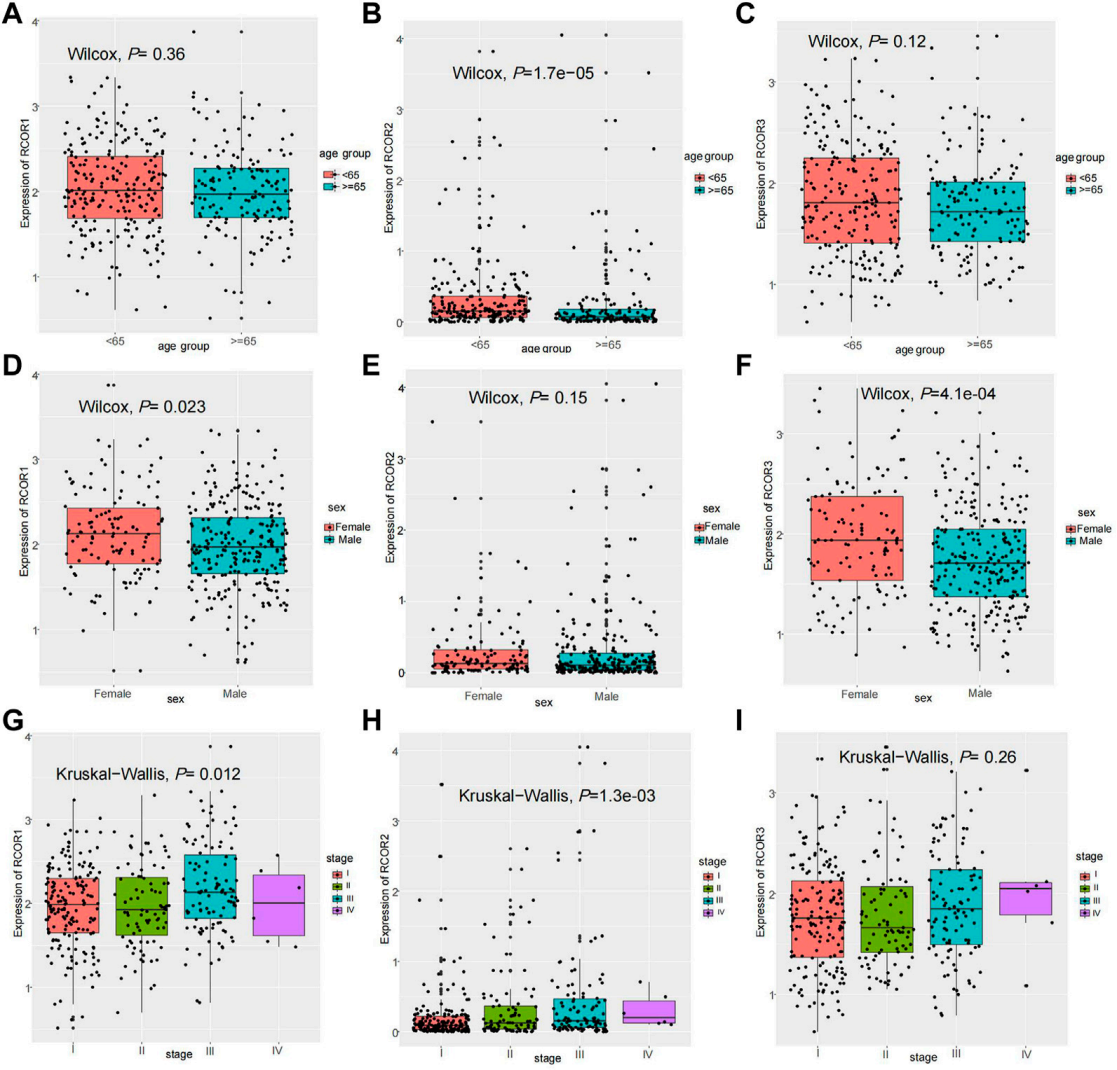
Clinical features analysis in HCC. Expression of *RCOR1* **(A)**, *RCOR2* **(B)** and *RCOR3* **(C)** in different age groups. Expression of *RCOR1* **(D)**, *RCOR2* **(E)** and *RCOR3* **(F)** in different sex. Expression of *RCOR1* **(G)**, *RCOR2* **(H)** and *RCOR3* **(I)** at different tumor stages.

According to *RCOR*s expression in HCC, correlation analysis with clinical features such as age, sex and tumor stage was conducted. Box plots demonstrated that *RCOR2* expression in HCC patients with age <65 was significantly higher than in age ≥65 group. *RCOR1* and *RCOR3* expression also seemed higer in age <65 groups without statistical significance (Figures 7A–C). *RCOR1* and *RCOR3* were significantly upregulated in HCC female samples (Figures 7D–F). *RCOR1* and *RCOR2* expression differed significantly among tumor stages, whereas no significance exists between *RCOR3* expression and tumor stages, we still noticed that *RCOR3* expression elevated progressively among stage II, III, and IV (Figures 7G–I). Taken together, these results revealed that *RCORs* expression was significantly correlated with cliniclpathological parameters in HCC.

### 3.7 Correlation between *RCOR*s expression and immune infiltration, immune checkpoints and stemness of HCC

**FIGURE 8:**
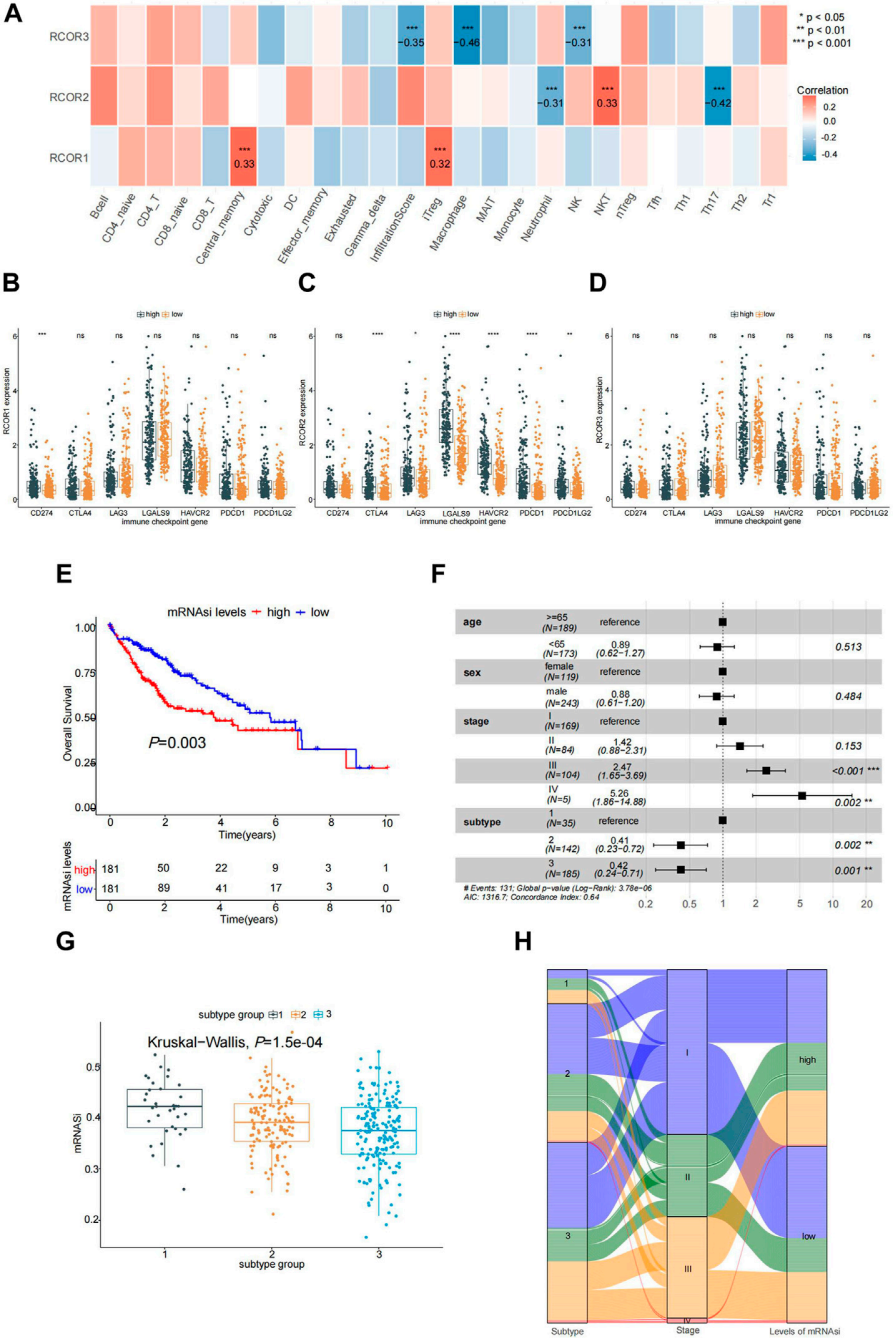
Association of *RCOR*s with HCC. **(A)** Correlation between *RCOR*s and immune infiltration from GSCA database; Expression of immune checkpoint moleculars between differentially expressed groups of *RCOR1* **(B)**, *RCOR2* **(C)** and *RCOR3* **(D)** in HCC; **(E)** Survival analysis between high and low HCC stemness groups; **(F)** Multivariate cox regression analysis; **(G)** Stemness indices among different molecular subtypes; **(H)** Relationship in tumor stage, subtype and stemness status. *p < 0.05, **p < 0.01, and ***p < 0.001.

To estimate the correlation of *RCOR*s expression with 24 immune cell types infiltration, “Immune” module of GSCA online tool was used. In Figure 8A, significant positive correlations were presented between *RCOR1* expression and infiltration of central memory T cell and iTreg (*p <* 0.05). *RCOR2* was negatively correlated with Neutrophil and Th17 cells infiltration, but positively with NKT cell (*p <* 0.05). *RCOR3* was negatively correlated with infiltration score, macrophage and NK cell (*p <* 0.05). To determine whether there is association between *RCOR* members and some immune checkpoint genes, we categorized the HCC samples into high and low groups in terms of median expression value of *RCOR*s, then compared the expression of immune checkpoint molecules (CD274, CTLA-4, LAG-3, LGALS9, HAVCR2, PDCD1, PDCD1LG2) between different groups of *RCOR*s. Figure 8B showed that high *RCOR1* group appeared to upregulate CD274 (*p <* 0.05), whereas no significance was seen on other immune checkpoint molecules. Conversely, *RCOR2* favorably correlated with all immune checkpoint molecules except CD274 (Figure 8C). However, none of these immune checkpoints demonstrated significant difference between high and low groups of *RCOR3* (Figure 8D).

According to *RCOR*s expression, HCC stemness indices were also calculated. Results can be seen in Supplementary Table S6. Samples were divided into two groups based on stemness indices. As a result, higher stemness indices group of *RCOR*s correlated with poor OS (*p <* 0.05) (Figure 8E). In addition, multivariate Cox regression analysis was conducted, where the mRNAsi group was removed through PH test (Supplementary Figure S9). As displayed in Figure 8F, significance can be found in tumor stage (Ⅲ, Ⅳ), tumor subtype (2, 3) in this model. Meanwhile, stemness indices among cancer molecular subtypes were compared by Kruaskal-wallis test (Figure 8G). Sankey diagram was used to display the relationship of stages, subtypes and stemness (Figure 8H).

### 3.8 Construction of regulatory networks of *RCOR*s in HCC

Upstream miRNAs of *RCOR*s were extracted from Mirecords, Mirtarbase and Tarbase databases and further retrieval was achieved by R package “multiMiR”. As a result, 177 RCORs-miRNA pairs were obtained. Then, we retrieved 462 miRNA-lncRNA pairs also by “multiMiR”, a ceRNA network was established through cytoscope, as shown in Supplementary Figure S10A. Subsequently, we downloaded 1,665 human transcription factor (TF) data from “AnimalTFDB” database, and 1,614 TFs were extracted from the TCGA-LIHC expression profile. By calculating the Pearson correlation coefficient between TFs and *RCOR*s expression, 304 *RCOR*s-TFs pairs were captured. The RCORs-TFs regulatory networks such as *RCOR1*-SP3, *RCOR3*-YY1 were shown in Supplementary Figure S10B. X2Kgui was used to predict the upstream kinases of *RCOR*s, a sum of 138 *RCOR*s-kinase pairs were acquired. The *RCOR*s-kinase regulatory networks were demonstrated in Supplementary Figure S10C, including *RCOR1*-ABL1, *RCOR2*-NLK, *RCOR3*-CDK2, etc. Totally, a comprehensive kinase-TF-ceRNA networks were constructed, comprising FRK-SP1-*RCOR1*-hsa-miR-520f-3p-TMEM105, PRKCZ-MYNN-*RCOR3*-hsa-miR-26b-5p-SNHG17 and so on (Supplementary Figure S10D).

### 3.9 RCOR1 is elevated in HCC and promotes HCC cell proliferation by inhibiting cell cycle arrest and cell apoptosis

**FIGURE 9:**
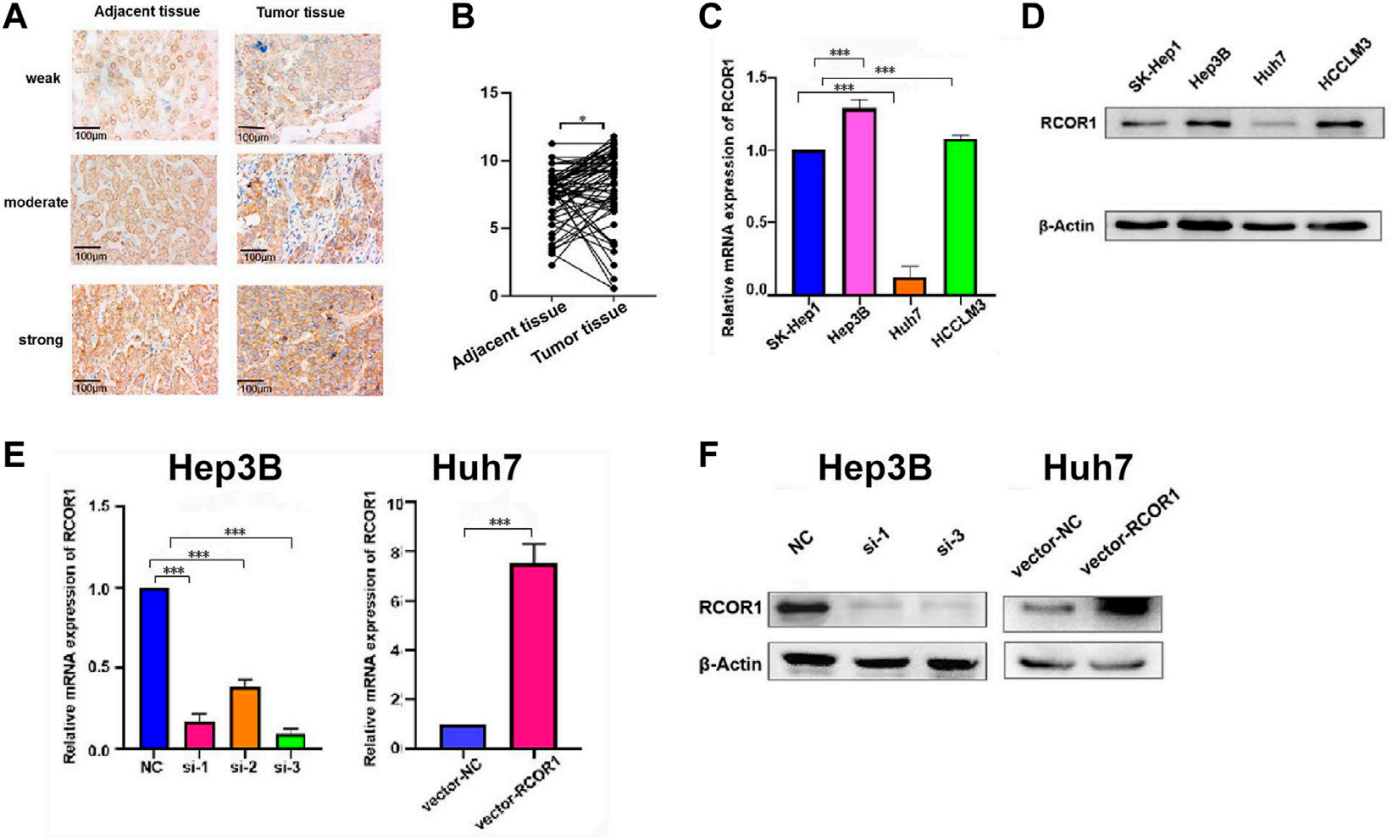
RCOR1 expression in HCC. **(A)** IHC staining of RCOR1 levels using HCC tissue microarrays; **(B)** IHC scoring of RCOR1 in tumor and adjacent non-tumor tissues; Expression of *RCOR1* in HCC cell lines at mRNA **(C)** and protein **(D)** levels. Knock-down and overexpression efficiency in Hep3B and Huh7 cells at mRNA **(E)** and protein **(F)** levels. *p < 0.05, **p < 0.01, and ***p < 0.001.

Since *RCOR1* is the only member of the *RCORs* family which showed significant difference both in sex and TNM stage, we chose *RCOR1* for further experiment. To detect the expression of RCOR1 in HCC tissue, we performed IHC staining of HCC tissue microarrays, indicating that RCOR1 protein was located in both nucleus and cytoplasm, and RCOR1 protein level was higher in tumor specimens than in matched adjacent non-tumor tissues (Figures 9A, B). *RCOR1* mRNA level in 4 HCC cell lines (SK-Hep1, Hep3B, Huh7, and HCCLM3) was also investigated. Compared with SK-Hep3 cells, *RCOR1* was significantly upregulated in Hep3B and HCCLM4, whereas it appeared downregulation in Huh7 (Figure 9C). The results were confirmed by detections in protein (Figure 9D). Therefore, Hep3B was selected for siRNA studies, and Huh7 was for overexpression researches. Their efficiency were verified by qPCR and western blotting respectively (Figures 9E, F). Si-3 were chosen for subsequent loss-of-functions analysis, and vector-*RCOR1* was for gain-of-function analysis.

**FIGURE 10:**
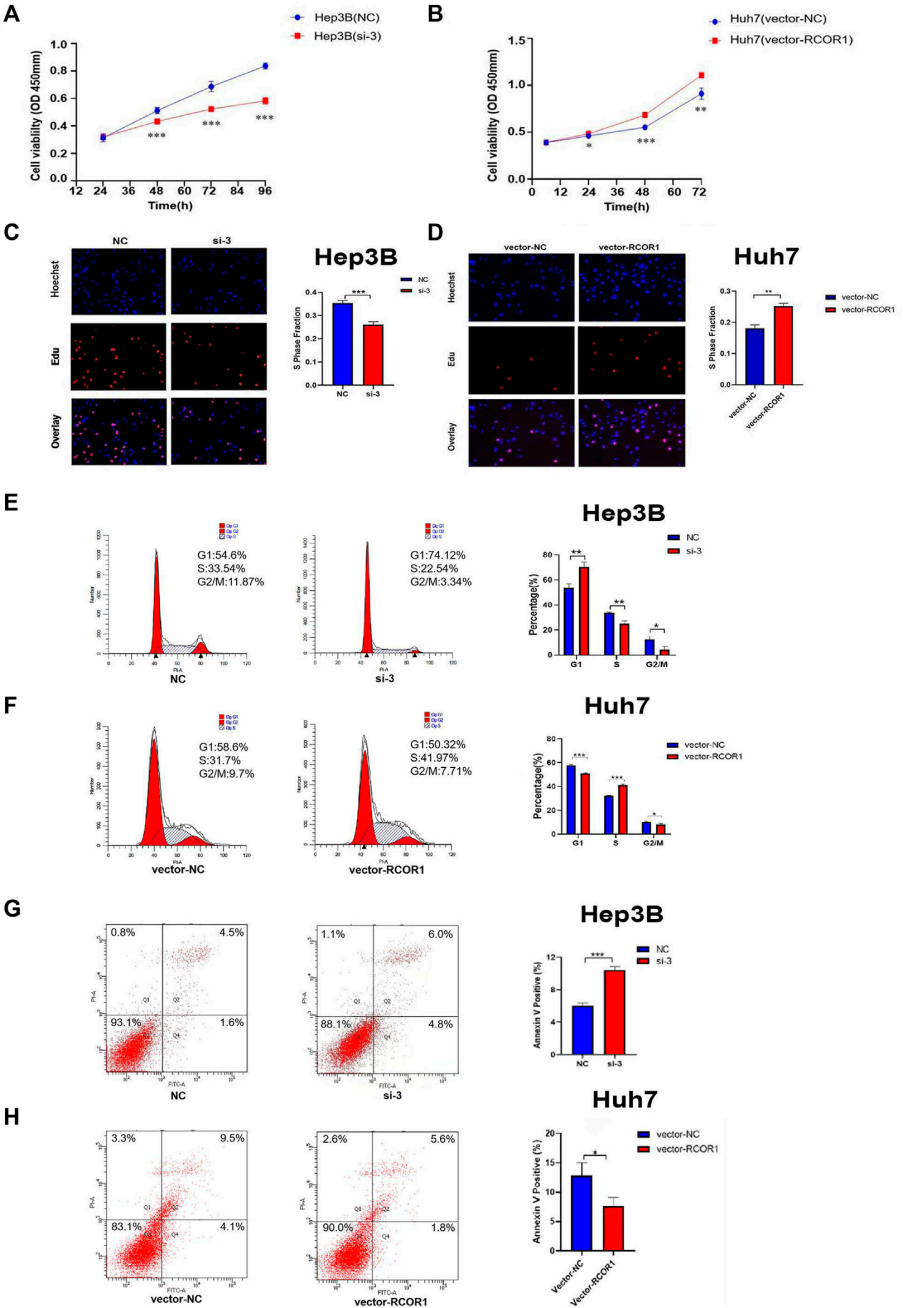
RCOR1 promotes cell growth in HCC cells. CCK8 assay in Hep3B **(A)** and Huh7 **(B)** cell line. EDU assays in Hep3B **(C)** and Huh7 cells **(D)**. Effect of RCOR1 on cell cycle in Hep3B **(E)** and Huh7 **(F)** cells, Apoptosis analysis of RCOR1 in Hep3B **(G)** and Huh7 **(H)** cells. *p < 0.05, **p < 0.01, and ***p < 0.001.

CCK-8 and EDU assays were used to characterize *RCOR1* effects on HCC cell proliferation, knockdown of *RCOR1* can significantly inhibit cell viability compared with siRNA-negative control (NC) group (Figures 10A, C), whereas upregulation of *RCOR1* raised cell viability (Figures 10B, D). Flow cytometry was used to analyze cell cycle and apoptosis. In *RCOR1*-knockdown Hep3B cells, the percentage of cells in the G1 phase increased while the percentage of cells in the S phase decreased significantly (Figure 10E). Compared with si-NC cells, annexin V positive cells in si-*RCOR1* Hep3B cells increased significantly (Figure 10G). The percentage of cells in the G1 phase decreased and the S phase cell percentage increased significantly in vector-*RCOR1* Huh7 cells compared to vector-NC cells (Figure 10F) and vector-*RCOR1* groups showed remarkably lower proportion of Annexin V positivity than that in vector-NC groups (Figure 10H).

## 4 Discussion

*RCOR*s are generally considered as components of silencing transcription factor REST. They have crucial function in regulating neuro-development, and mediating neuron biological processes in a REST-independent way (Maksour et al., 2020). Moreover, a few evidences showed that *RCOR*s produce marked effects on various disease. For example, *RCOR*s were considered to be involved in the terminal differentiation of OA chondrocytes (Xiao et al., 2010). In recent years, an increasing number of studies revealed that *RCOR*s might play a role in the process of tumorigenesis. So far, there is no systematic analysis of *RCOR*s in cancers. Our study elaborated the tumor-related features of *RCOR* family genes in multiple dimensions.

GF60-mutant *RCOR1* was isolated in drosophila melanogaster follicular epithelium and activated Notch signaling pathway (Domanitskaya and Schüpbach, 2012). In our study, genetic alteration of *RCOR*s in pan-cancer was detected, revealing that the alteration status of *RCOR*s has a significant impact on the survival rate of cancer patients. The relatively highly potential anti-neoplastic drugs that associated to *RCOR*s were screened in this study, including RDEA119, Trametinib, Selumatinib. They are all belong to selective MEK1/MEK2 inhibitor, which can activate cancer cell autophagy, inhibiting cell proliferation, migration and inducing apoptosis. This indicated that *RCOR*s probably regulate tumorgenesis through MAPK signaling pathway. Thus, drug sensitivity analysis of *RCOR*s can offer more clues for further signaling pathway researches in cancers. GSCALite analysis showed that each member of *RCOR*s exhibited different activation or inhibition in common signaling pathways, indicating that *RCOR1*, *RCOR2*, *RCOR3* probably work independently or competitively in tumor progression so that leading to opposite effect on the prognosis of cancer patients. Visible inhibition of Hormone ER couid be seen significantly associated with *RCOR1* and *RCOR2*, whereas activation of this signaling pathway strongly associated with *RCOR3*. Recently, Martinez et al. has come up with that *RCOR*s (CoREST) drive the tumorgenesis of ER + breast cancer and induce resistantance to endocrine therapy by switching the recruiting site of the complex (Garcia-Martinez et al., 2022). Consistent of our study, there is an imagination that *RCOR*s are able to function as potential targets of advanced breast cancer treatment.

Several studies clarified that *RCOR*s may be effective in tumor immunity. Xiong et al. (2020) proved that the conditional deletion of *RCOR1* in Foxp3^+^ Tregs had waken the function of Tregs, while the proportion of IL-2 and IFN-γraised up in peripheral lymphoid tissues and promoted antitumor immunity. In our study, expression of *RCOR*s correlated to the infiltration of congenital and specific immune cells in HCC. Given that, it’s worthwhile to detect the tumorous immune characteristics of *RCOR* genes.

Emerging of competitive endogenous RNA (ceRNA) has started a new form of gene expression regulation, which is composed of mRNA, pseudogenes encoding genes, long chain non coding RNA (lncRNA) and miRNA (Salmena et al., 2011). Compared with miRNA regulation network, it is more sophisticated and complex, which provides a more detectable perspective for researchers to conduct transcriptome research, and is conducive to a more in-depth and comprehensive explanation of biological phenomena in cancer. Here, we constructed ceRNA regulatory networks by directly retrieving the miRNAs that were verified related to *RCOR*s and the lncRNAs which were related to miRNAs. A large account of researches has illustrated that *RCOR*s were targeted by microRNA such as miR-22 (Volvert et al., 2014), miR-124 (Baudet et al., 2011), miR9* (Packer et al., 2008), miR-432 (Das and Bhattacharyya, 2014) etc., and played crucial role in many biological processes. According to our study, in HCC, we extracted *RCOR*s-miRNA-lncRNA pairs, such as *RCOR1*-hsa-miR-9-3p-CT62, *RCOR2*-hsa-miR-1343-3p-LINC02693, *RCOR3*-hsa-miR-128-3p, etc., offering potential targets for cancer therapy. Besides, based on the silencing effect on transcription of *RCOR*s, we also filtered transcription factors (TF), which might be the potential targets in occurrence and development of HCC. Our study captured 304 TF-mRNA pairs in *RCOR1* and *RCOR3*. There is no effective discovery in *RCOR2,* lack of detailed researches. More evidences are needed to reveal how *RCOR*s affect TF in cancer progression. Zhang et al. (2015) declared that PLK1 kinase reduced levels of *RCOR*s through degradation of ZNF198 in HBV-replicating cells, which might induce the process of liver carcinogenesis. In our study, we predicted the upstream kinases of *RCOR*s, obtained 138 RCORs-kinase pairs, and constructed *RCOR*s-kinase regulatory networks. Totally, we drew the Kinase-TF-ceRNA regulatory networks of HCC, which systematically demonstrated the potential regulation landscape.

In signaling pathway analysis, Apoptosis and Cell cycle pathways seemed visibly activated by *RCOR1* and *RCOR2*, whereas *RCOR3* presented evident inhibition in these two pathways. Based on this observation, we consider *RCOR*s probably regulate tumor growth through mediating cell cycle and/or apoptosis. Our study verified RCOR1 enhance HCC cell growth by affecting cell cycle and cell apoptosis. Deregulation of cell cycle is regarded as one of the common causalities of the early steps of hepatocarcigenesis (Greenbaum, 2004). Our results demonstrated that RCOR1 play a role in regulating G1/S cell cycle phase in HCC cells according to cell cycle flow-cytometry analysis. Lack of apoptosis is related to the development and progression of liver tumors (Fabregat et al., 2007). Here we also proved overexpressed RCOR1 induce cell apoptosis in HCC cells. Above all, our current study suggested that RCOR1 regulates cell cycle and apoptosis, thereby promoting the growth of HCC cells. However, further studies especially detailed mechanism researches and *in-vivo* studies are needed in the near future to further explore and validate the role of *RCORs* family in HCC.

In conclusion, our findings systematically illustrated the potential molecular mechanisms of *RCOR*s in pan-cancer, offering a benchmark for cancer-related research. Moreover, RCOR1 acts as an oncogene in HCC and promotes the proliferation of HCC cells by inhibiting cell cycle arrest and cell apoptosis.

## Data Availability

The datasets presented in this study can be found in online repositories. The names of the repository/repositories and accession number(s) can be found in the article/Supplementary Material.
