# Supplementary material for: Comprehensive analysis of REST corepressors (RCORs) in pan-cancer
Source: Front Cell Dev Biol. 2023 Jun 5;11:1162344. doi: 10.3389/fcell.2023.1162344 (PMC10277624; doi:10.3389/fcell.2023.1162344)

**Supplementary Figure 10.** Regulatory network of *RCOR*s in HCC. (**A**) *RCOR*s-ceRNA regulatory network, (**B**) TF-*RCOR*s regulatory network, (**C**) *RCOR*s-kinase regulatory network, (**D**) kinase-TF-ceRNA regulatory network.

**A**


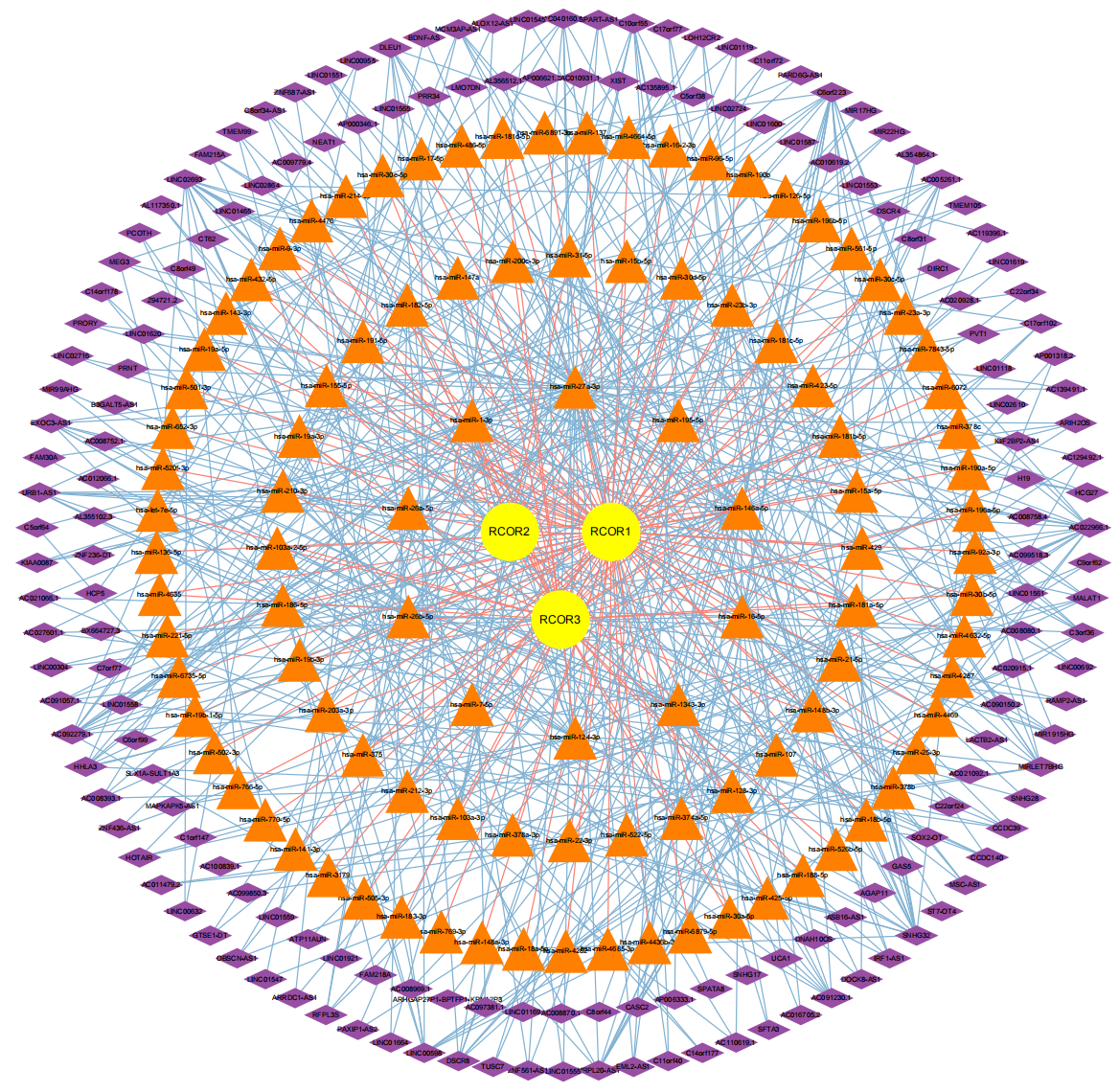


**B**


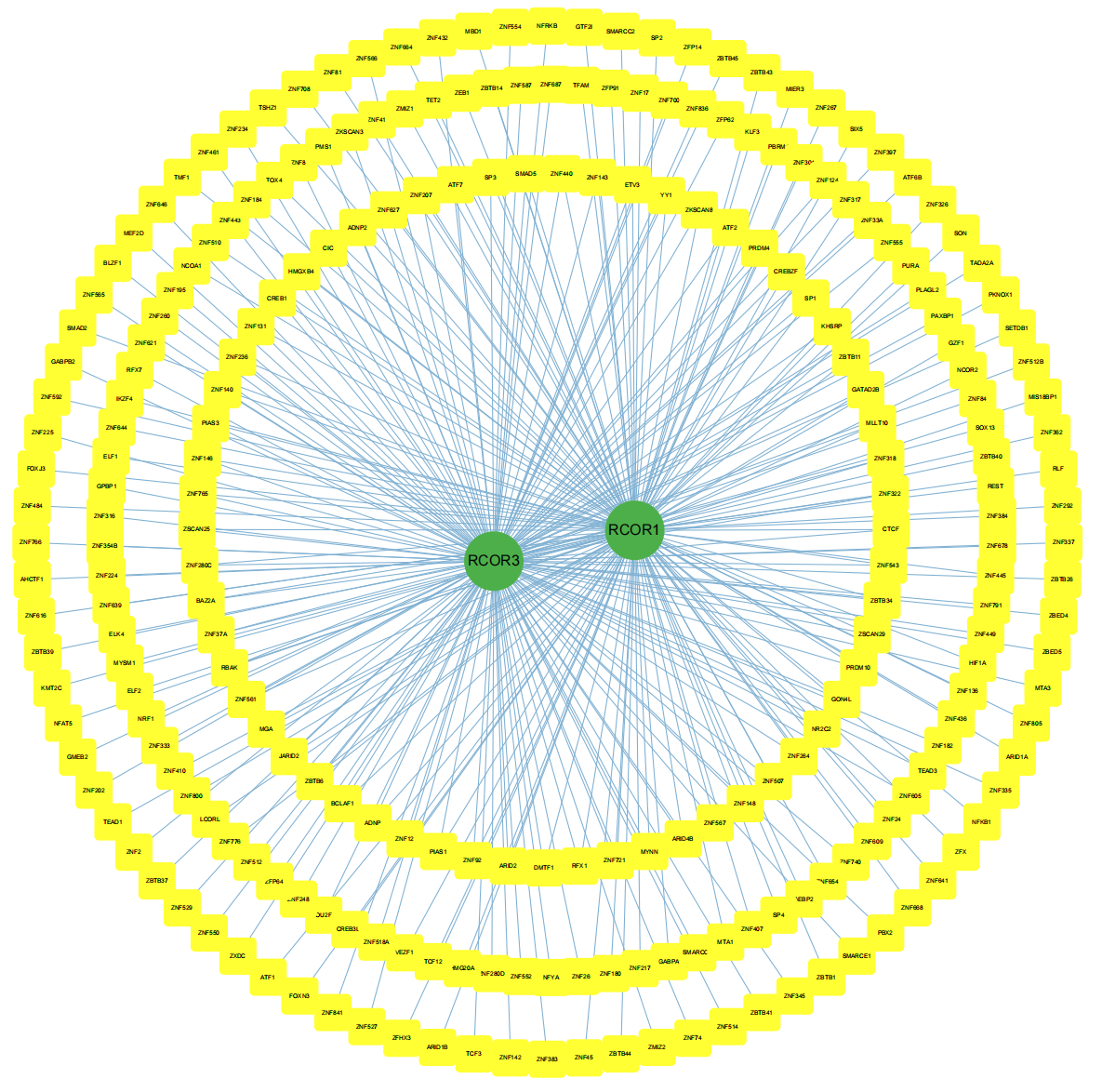


**C**


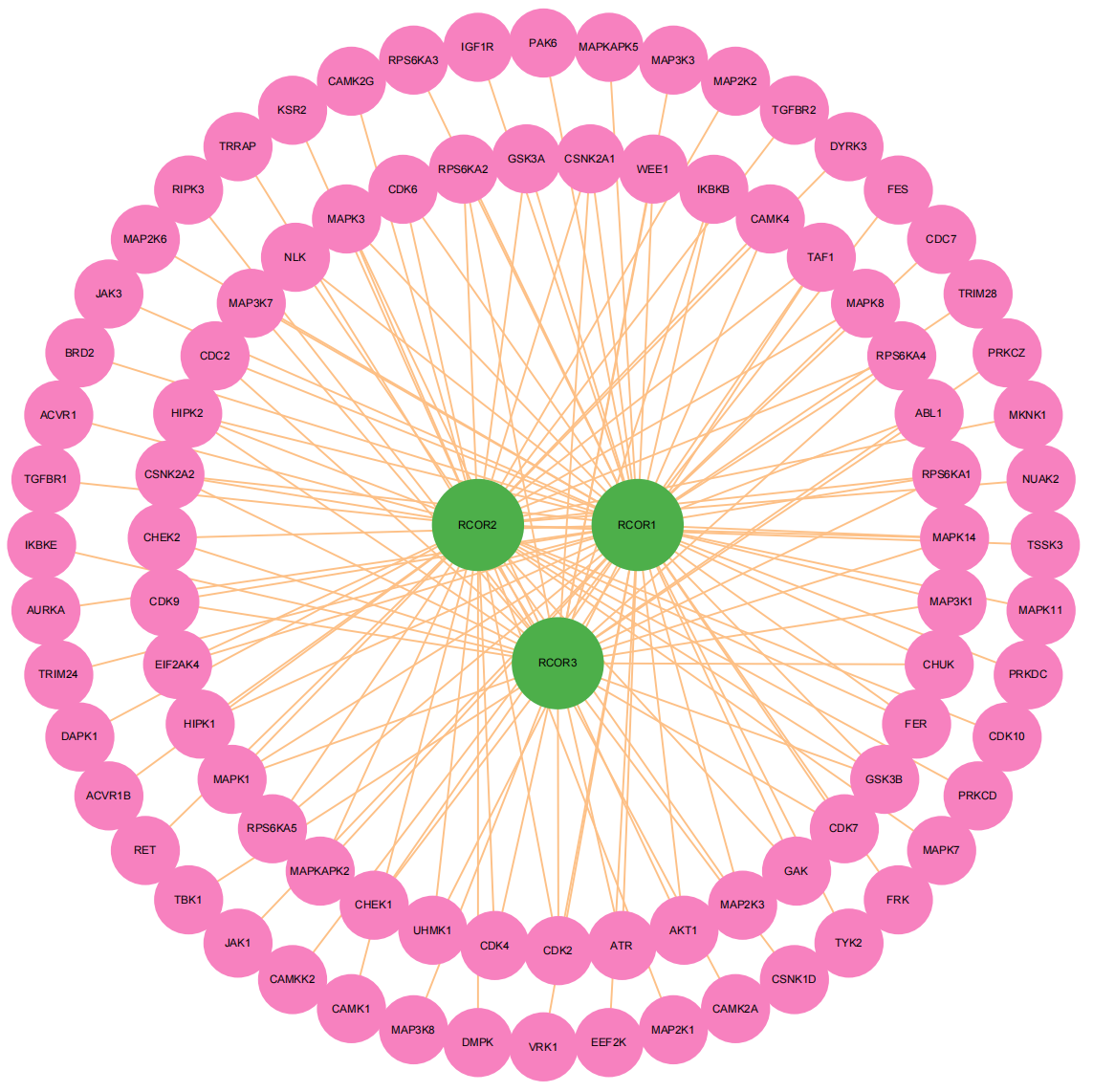


**D**


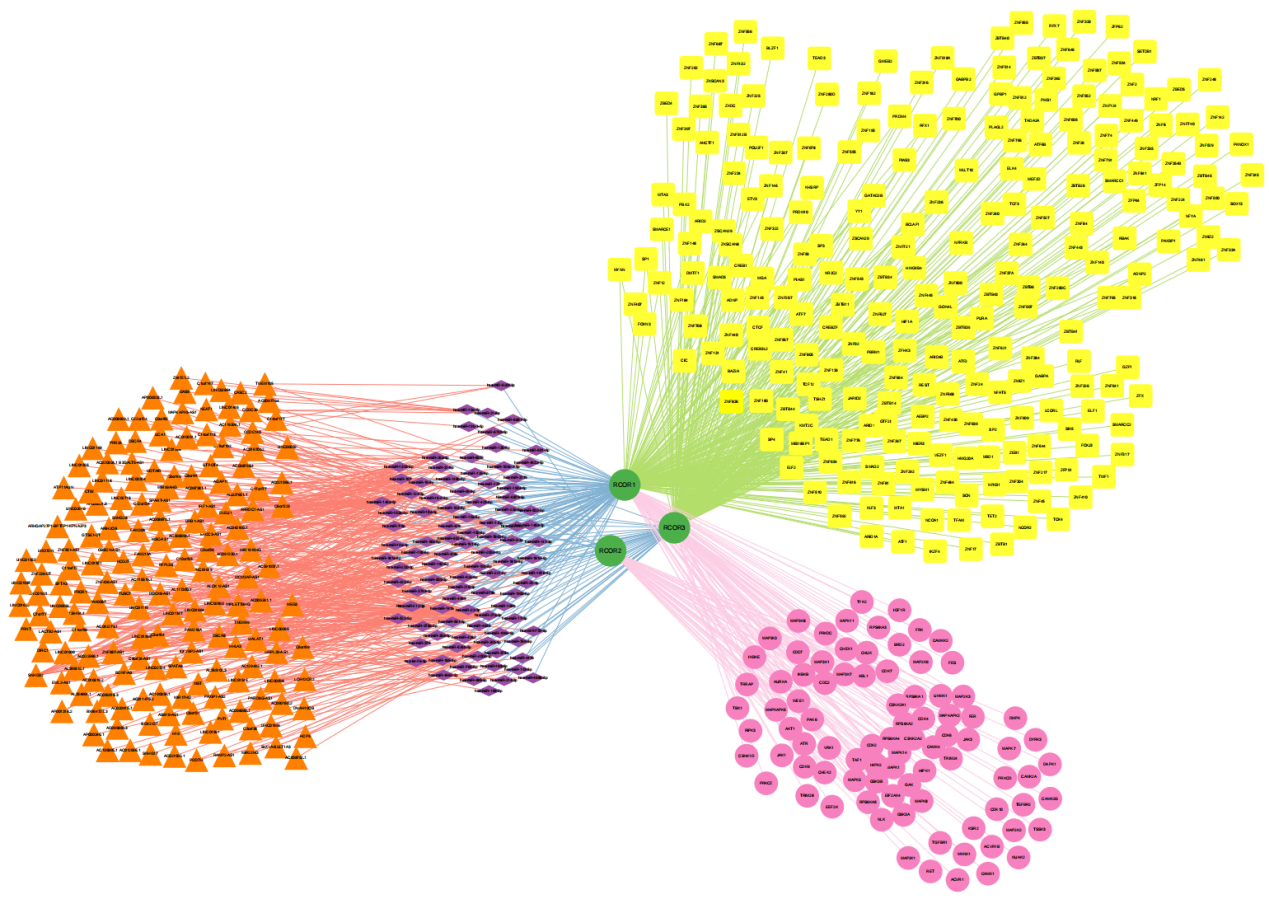

Supplement: Supplementary file 1 [file DataSheet1.zip › Supplementary Material/Supplementary Figure 10.DOCX]
