# Supplementary material for: Comprehensive analysis of REST corepressors (RCORs) in pan-cancer
Source: Front Cell Dev Biol. 2023 Jun 5;11:1162344. doi: 10.3389/fcell.2023.1162344 (PMC10277624; doi:10.3389/fcell.2023.1162344)
Supplement: Supplementary file 1 [file DataSheet1.zip › Supplementary Material/Supplementary Figure 5.DOCX]

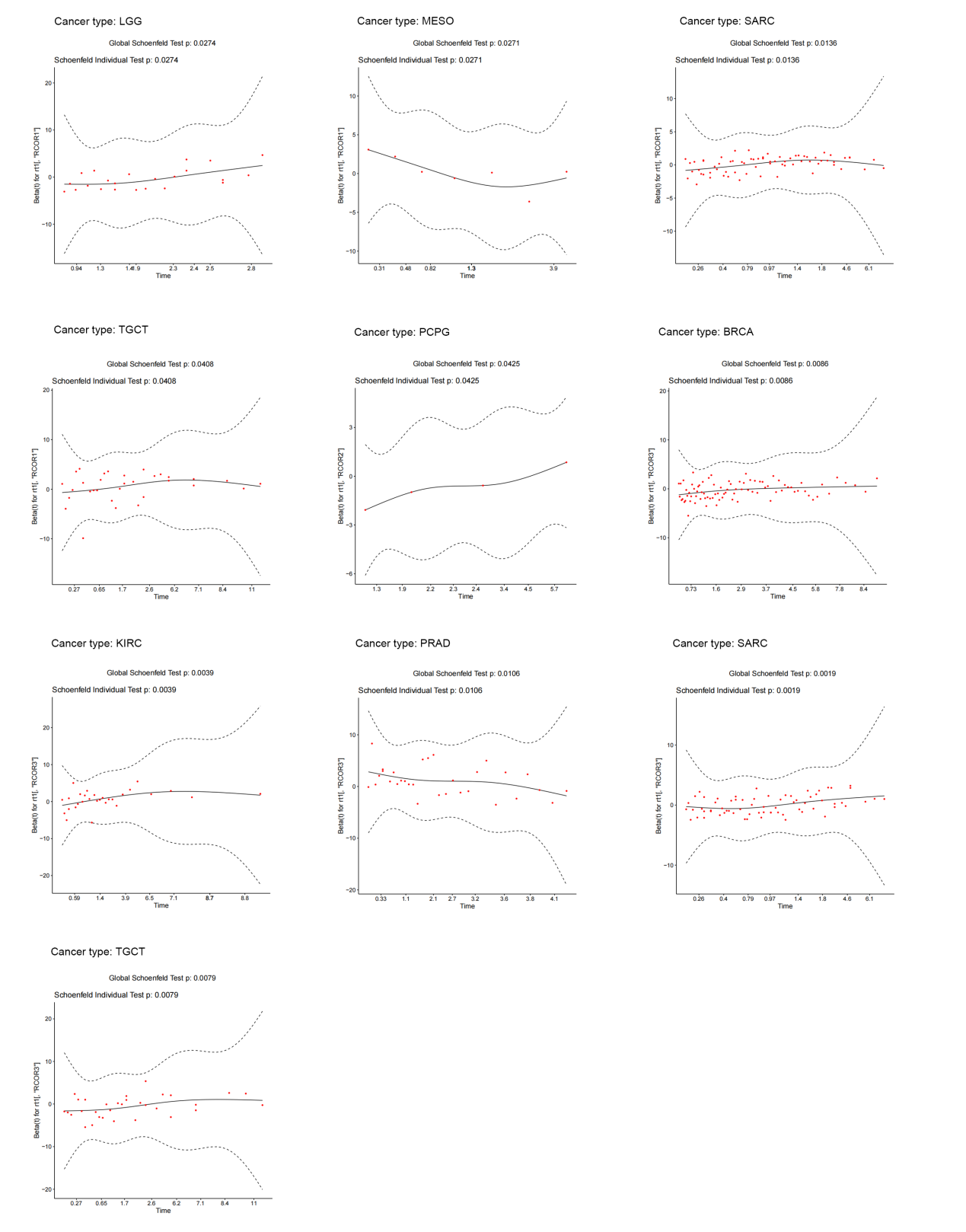


**Supplementary Figure 5.** The proportional hazards (PH) assumption test of the univariate Cox proportional hazards regression models of *RCOR*s among various cancers. The survival information contained disease-free survival (DFS). Only unsatisfied results were indicated.
