# Supplementary material for: Comprehensive analysis of REST corepressors (RCORs) in pan-cancer
Source: Front Cell Dev Biol. 2023 Jun 5;11:1162344. doi: 10.3389/fcell.2023.1162344 (PMC10277624; doi:10.3389/fcell.2023.1162344)
Supplement: Supplementary file 1 [file DataSheet1.zip › Supplementary Material/Supplementary Figure 7.DOCX]

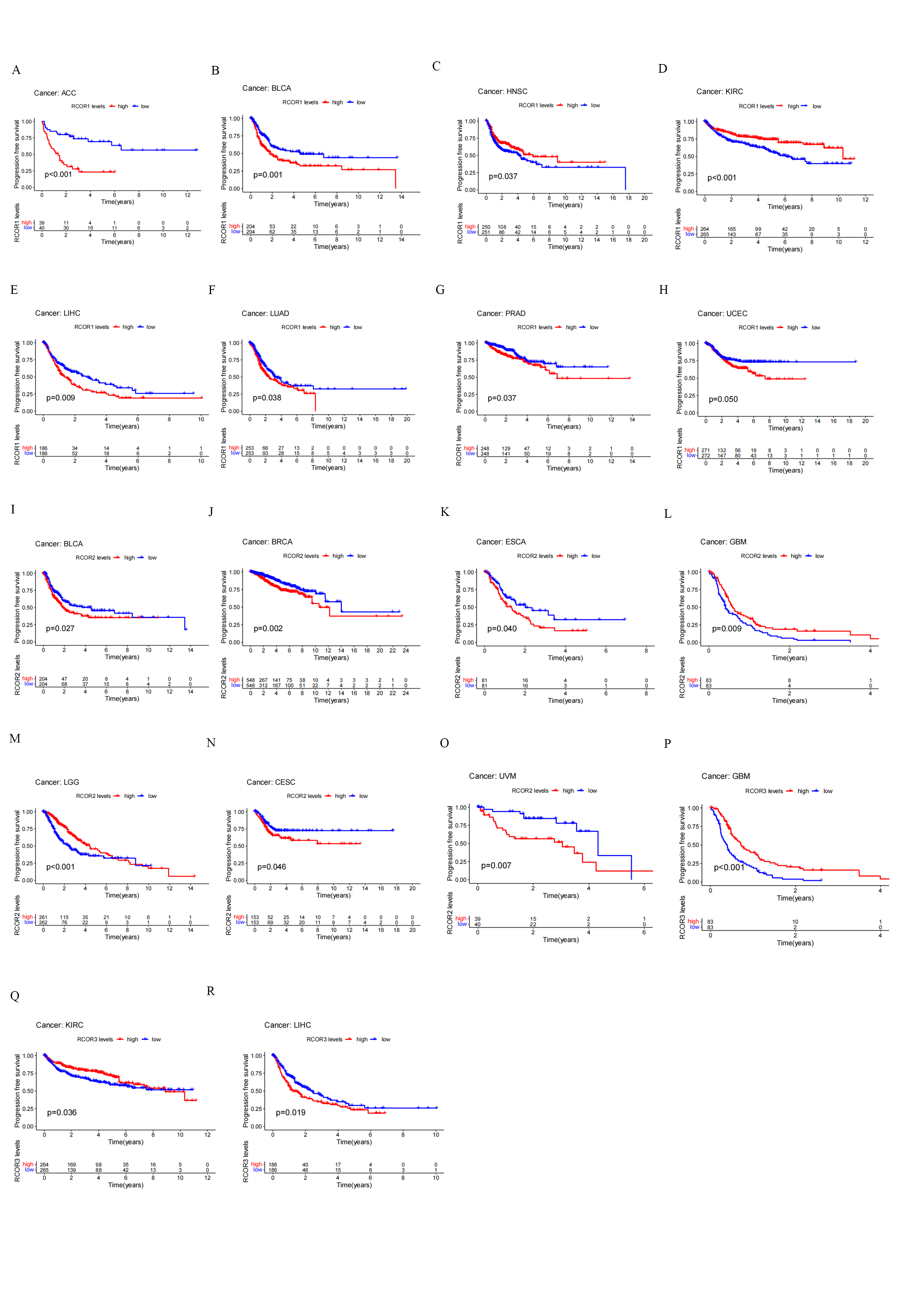


**Supplementary Figure 7.** PFS survival curves of *RCOR*s in different cancers. *RCOR1* is correlated with PFS in ACC (**A**), BLCA (**B**), HNSC (**C**) , KIRC (**D**) , LIHC (**E**) , LUAD (**F**) , PRAD (**G**) and UCEC (**H**). *RCOR2* is related to BLCA (**I**), BRCA (**J**), ESCA(**K**), GBM (**L**) , LGG (**M**), CESC (**N**) and UVM (**O**). *RCOR3* is associated with GBM (**P**), KIRC (**Q**) and LIHC (**R**). Only significant differences were indicated in the results.
