# Supplementary material for: Comprehensive analysis of REST corepressors (RCORs) in pan-cancer
Source: Front Cell Dev Biol. 2023 Jun 5;11:1162344. doi: 10.3389/fcell.2023.1162344 (PMC10277624; doi:10.3389/fcell.2023.1162344)
Supplement: Supplementary file 1 [file DataSheet1.zip › Supplementary Material/Supplementary Figure 8.DOCX]

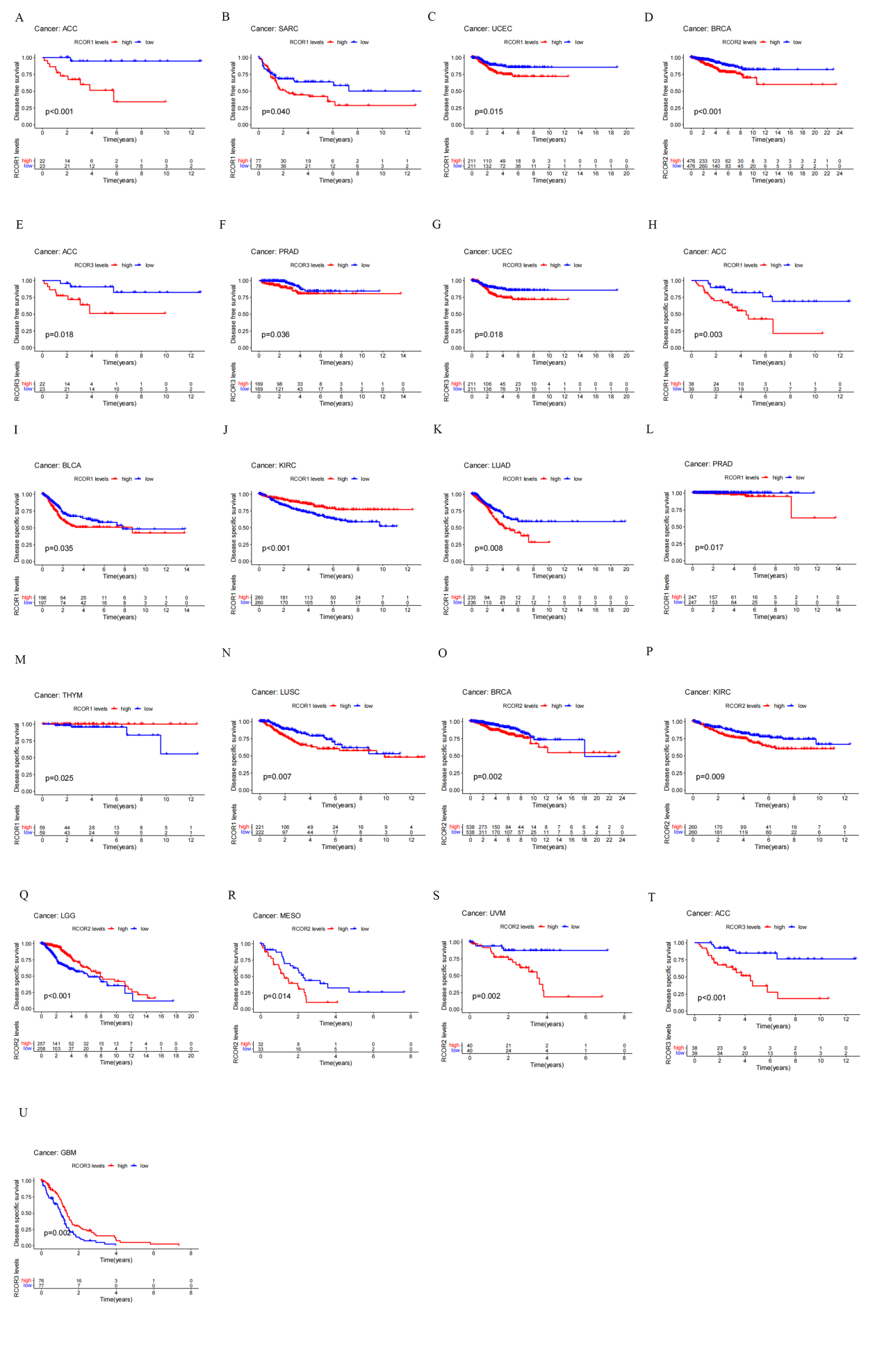


**Supplementary Figure 8.** DFS and DSS survival curves of *RCOR*s in different cancers. *RCOR1* is predictable for DFS in ACC (**A**), SARC (**B**) and UCEC (**C**) , while is predictable for DSS in ACC (**H**) , BLCA (**I**), KIRC (**J**) , LUAD (**K**) , PRAD (**L**), THYM (**M**) and LUSC (**N**). *RCOR2* is predictable for DFS in BRCA (**D**); *RCOR3* is predictable for DFS in ACC (**E**), PRAD (**F**) and UCEC(**G**). *RCOR2* is predictable for DSS in BRCA (**O**) , KIRC (**P**), LGG (**Q**), MESO (**R**) and UVM (**S**). *RCOR3* is predictable for DSS in ACC (**T**) and GBM (**U**). Only significant differences were indicated in the results.
