# Supplementary material for: Comprehensive analysis of REST corepressors (RCORs) in pan-cancer
Source: Front Cell Dev Biol. 2023 Jun 5;11:1162344. doi: 10.3389/fcell.2023.1162344 (PMC10277624; doi:10.3389/fcell.2023.1162344)
Supplement: Supplementary file 1 [file DataSheet1.zip › Supplementary Material/Supplementary Figure 9.DOCX]

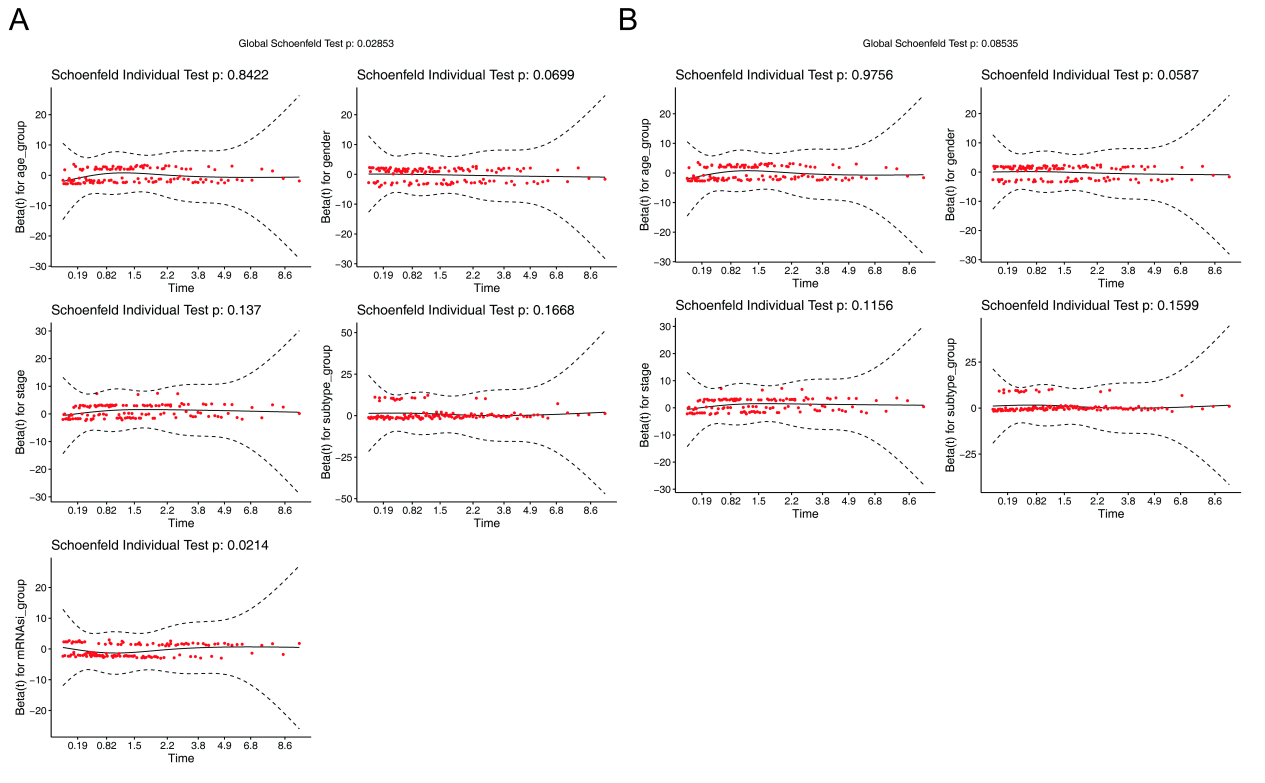


**Supplementary Figure 9.** The PH assumption test of the multivariate Cox proportional hazards regression models that was constructed based on the clinical features. The results before (**A**) and after (**B**) elimination of mRNAsi_group were exhibited.
