# Supplementary material for: Comprehensive analysis of REST corepressors (RCORs) in pan-cancer
Source: Front Cell Dev Biol. 2023 Jun 5;11:1162344. doi: 10.3389/fcell.2023.1162344 (PMC10277624; doi:10.3389/fcell.2023.1162344)
Supplement: Supplementary file 1 [file DataSheet1.zip › Supplementary Material/Supplementary Table 3 + 4.DOCX]

| **Supplementary Table 3. Correlation between clinicopatological parameters and different molecular subtypes in BRCA** | | | | | |
| --- | --- | --- | --- | --- | --- |
|  | Subtypes | | | | *P*-value |
|  | Ⅰ | Ⅱ | Ⅲ | Ⅳ |  |
| Number | 332 | 332 | 170 | 256 |  |
| Sex |  |  |  |  |  |
| Male(%) | 2(0.6) | 6 (1.8) | 2 (1.2) | 2 (0.8) | 0.47 |
| Female(%) | 330(99.4) | 326 (98.2) | 168 (98.8) | 254 (99.2) |  |
| Age (mean±SD) | 59.23 (±13.44) | 59.92 (±12.81) | 56.20 (±12.12) | 57.00 (±13.78) | 0.004**^**^** |
| T_stage (%) |  |  |  |  | 0.433 |
| T1 | 85 (25.6) | 94 (28.3) | 39 (22.9) | 61 (23.8) |  |
| T2 | 194 (58.4) | 181 (54.5) | 103 (60.6) | 153 (59.8) |  |
| T3 | 42 (12.7) | 46 (13.9) | 20 (11.8) | 29 (11.3) |  |
| T4 | 11 (3.3) | 10 (3.0) | 6 (3.5) | 13 (5.1) |  |
| Tx | 0 (0.0) | 1 (0.3) | 2 (1.2) | 0 (0.0) |  |
| M_stage (%) |  |  |  |  | 0.057 |
| M0 | 276 (83.1) | 269 (81.0) | 139 (81.8) | 223 (87.1) |  |
| M1 | 7 (2.1) | 5 (1.5) | 8 (4.7) | 2 (0.8) |  |
| MX | 49 (14.8) | 58 (17.5) | 23 (13.5) | 31 (12.1) |  |
| N_stage (%) |  |  |  |  | 0.264 |
| N0 | 149 (44.9) | 164 (49.4) | 88 (51.8) | 113 (44.1) |  |
| N1 | 105 (31.6) | 116 (34.9) | 52 (30.6) | 87 (34.0) |  |
| N2 | 45 (13.6) | 24 (7.2) | 16 (9.4) | 35 (13.7) |  |
| N3 | 24 (7.2) | 23 (6.9) | 13 (7.6) | 16 (6.2) |  |
| NX | 9 (2.7) | 5 (1.5) | 1 (0.6) | 5 (2.0) |  |
| stage (%) |  |  |  |  | 0.07 |
| I | 48 (14.5) | 65 (19.6) | 25 (14.7) | 43 (16.8) |  |
| II | 183 (55.1) | 191 (57.5) | 104 (61.2) | 143 (55.9) |  |
| III | 95 (28.6) | 71 (21.4) | 34 (20.0) | 68 (26.6) |  |
| IV | 6 (1.8) | 5 (1.5) | 7 (4.1) | 2 (0.8) |  |
| group (%) |  |  |  |  | <0.001**^***^** |
| 1 | 332 (100.0) | 0 (0.0) | 0 (0.0) | 0 (0.0) |  |
| 2 | 0 (0.0) | 332 (100.0) | 0 (0.0) | 0 (0.0) |  |
| 3 | 0 (0.0) | 0 (0.0) | 170 (100.0) | 0 (0.0) |  |
| 4 | 0 (0.0) | 0 (0.0) | 0 (0.0) | 256 (100.0) |  |

| **Supplementary Table 4. Correlation between clinicopatological parameters and different molecular subtypes in BLCA** | | | | |
| --- | --- | --- | --- | --- |
|  | Subtypes | | | *P*-value |
|  | Ⅰ | Ⅱ | Ⅲ |  |
| Number | 138 | 118 | 152 |  |
| Sex |  |  |  |  |
| Male(%) | 96(69.6) | 97(82.2) | 108(71.1) | 0.046**^*^** |
| Female(%) | 42(30.4) | 21(17.8) | 44(28.9) |  |
| Age(mean±SD) | 66.38(±10.97) | 70.07(±10.10) | 68.09(±10.45) | 0.021**^*^** |
| T_stage (%) |  |  |  | 0.651 |
| T0 | 1 (0.8) | 0 (0.0) | 0 (0.0) |  |
| T1 | 1 (0.8) | 0 (0.0) | 2 (1.4) |  |
| T2 | 44 (36.1) | 31 (29.0) | 44 (29.9) |  |
| T3 | 59 (48.4) | 58 (54.2) | 77 (52.4) |  |
| T4 | 16 (13.1) | 18 (16.8) | 24 (16.3) |  |
| Tx | 1 (0.8) | 0 (0.0) | 0 (0.0) |  |
| M_stage (%) |  |  |  | 0.07 |
| M0 | 77 (56.2) | 54 (45.8) | 65 (43.0) |  |
| M1 | 2 (1.5) | 6 (5.1) | 3 (2.0) |  |
| MX | 58 (42.3) | 58 (49.2) | 83 (55.0) |  |
| N_stage (%) |  |  |  | 0.47 |
| N0 | 80 (58.8) | 71 (61.2) | 86 (57.3) |  |
| N1 | 13 (9.6) | 10 (8.6) | 23 (15.3) |  |
| N2 | 26 (19.1) | 20 (17.2) | 29 (19.3) |  |
| N3 | 2 (1.5) | 2 (1.7) | 4 (2.7) |  |
| NX | 15 (11.0) | 13 (11.2) | 8 (5.3) |  |
| stage (%) |  |  |  | 0.498 |
| I | 1 (0.7) | 0 (0.0) | 1 (0.7) |  |
| II | 51 (37.0) | 37 (31.4) | 42 (27.6) |  |
| III | 44 (31.9) | 46 (39.0) | 52 (34.2) |  |
| IV | 42 (30.4) | 35 (29.7) | 57 (37.5) |  |
| group (%) |  |  |  | <0.001**^***^** |
| 1 | 138 (100.0) | 0 (0.0) | 0 (0.0) |  |
| 2 | 0 (0.0) | 118 (100.0) | 0 (0.0) |  |
| 3 | 0 (0.0) | 0 (0.0) | 152 (100.0) |  |
