# Supplementary figures and images for: Comprehensive analysis of REST corepressors (RCORs) in pan-cancer
Source: Front Cell Dev Biol. 2023 Jun 5;11:1162344. doi: 10.3389/fcell.2023.1162344 (PMC10277624; doi:10.3389/fcell.2023.1162344)

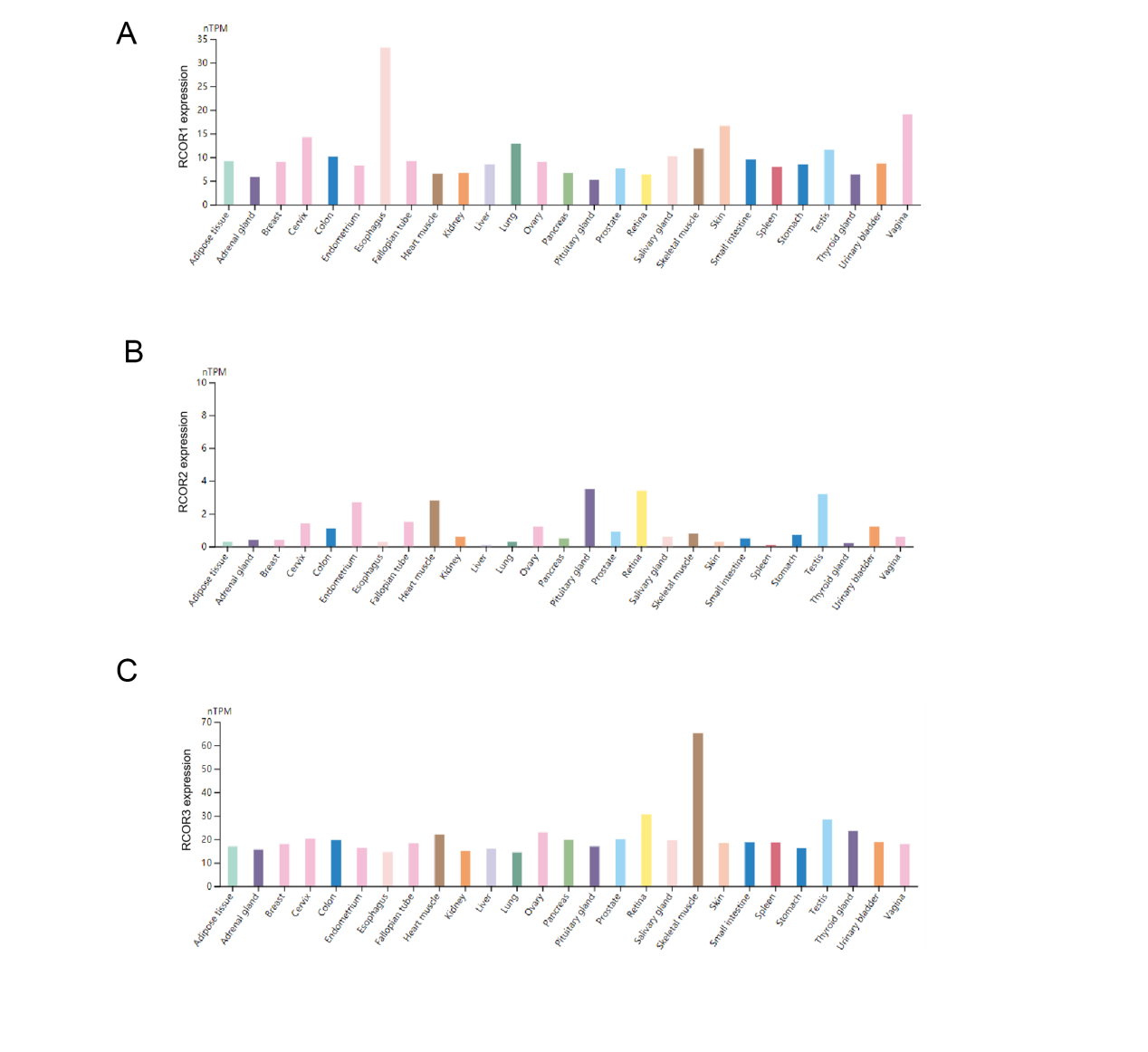


**Supplementary Figure 1.** *RCOR1* (**A**) , *RCOR2* (**B**) and *RCOR3* (**C**) expression among different normal samples.

Supplement: Supplementary file 1 [file DataSheet1.zip › Supplementary Material/Supplementary Figure 1.DOCX]

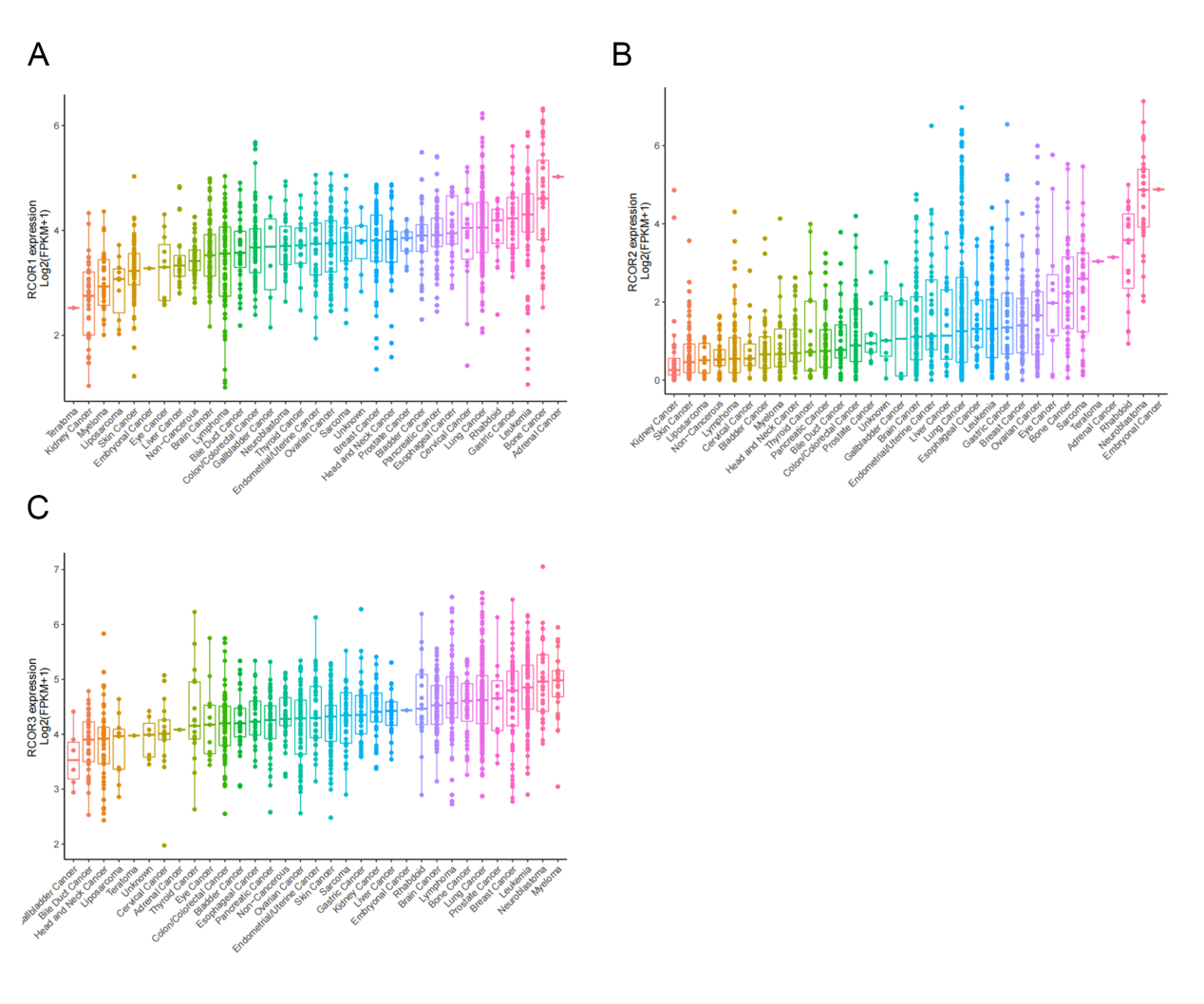


**Supplementary Figure 2.** *RCOR1* (**A**) , *RCOR2* (**B**) and *RCOR3* (**C**) expression in various cell lines.

Supplement: Supplementary file 1 [file DataSheet1.zip › Supplementary Material/Supplementary Figure 2.DOCX]
